# Developmental dynamics of mitochondrial mRNA abundance and editing reveal roles for temperature and the differentiation-repressive kinase RDK1 in cytochrome oxidase subunit II mRNA editing

**DOI:** 10.1128/mbio.01854-23

**Published:** 2023-10-05

**Authors:** Joseph T. Smith, Brianna Tylec, Arunasalam Naguleswaran, Isabel Roditi, Laurie K. Read

**Affiliations:** 1 Department of Microbiology and Immunology, Jacobs School of Medicine and Biomedical Sciences, University at Buffalo, Buffalo, New York, USA; 2 Institute of Cell Biology, University of Bern, Bern, Switzerland; University of Geneva, Geneva, Switzerland

**Keywords:** *Trypanosoma brucei*, RNA editing, differentiation, developmental regulation

## Abstract

Developmental regulation of mitochondrial uridine insertion/deletion editing in *Trypanosoma brucei* is necessary to modulate parasite metabolism as it shifts from dependence on glycolysis for ATP production in the mammalian bloodstream form (BSF) to oxidative phosphorylation in the insect procyclic form (PCF). However, the timing and stimuli that regulate mRNA editing have been poorly characterized. Here, we utilized a pleomorphic *T. brucei* strain and quantitative RT-PCR and droplet digital PCR analyses to evaluate the changes in total mRNA abundance and editing as parasites progressively differentiate from slender BSF to PCF and investigate the effect of individual stimuli on mitochondrial gene expression. We observed little change during the slender-to-stumpy BSF transition. Rather, we found that mainly the mitochondrial cytochrome (COI, COII, COIII, and CYb) mRNAs are upregulated within 24 h after stumpy BSF is stimulated to differentiate to PCF *in vitro* and during *in vivo* tsetse fly infections. Temperature reduction from 37°C to 27°C is a critical factor for increasing the editing of COII and COIII mRNAs and COIV protein expression but not the editing of CYb mRNA or RISP protein expression. We further demonstrate that the depletion of the differentiation-repressive kinase RDK1 couples with temperature reduction to stimulate COII mRNA editing, and the accessory factor p22 is required for the cold-responsive upregulation of COII mRNA editing. Overall, we show that cytochrome mRNAs are regulated during development by distinct stimuli through a variety of methods to increase their abundance and/or editing.

**IMPORTANCE:**

*Trypanosoma brucei* is the unicellular parasite that causes African sleeping sickness and nagana disease in livestock. The parasite has a complex life cycle consisting of several developmental forms in the human and tsetse fly insect vector. Both the mammalian and insect hosts provide different nutritional environments, so *T. brucei* must adapt its metabolism to promote its survival and to complete its life cycle. As *T. brucei* is transmitted from the human host to the fly, the parasite must regulate its mitochondrial gene expression through a process called uridine insertion/deletion editing to achieve mRNAs capable of being translated into functional respiratory chain proteins required for energy production in the insect host. Therefore, it is essential to understand the mechanisms by which *T. brucei* regulates mitochondrial gene expression during transmission from the mammalian host to the insect vector.

## INTRODUCTION

*Trypanosoma brucei* causes human African trypanosomiasis and nagana in domesticated animals and livestock (1, 2). This protozoan parasite has a complex life cycle involving both mammalian and tsetse fly hosts (3, 4) and progresses through a series of developmental stages with distinct transcriptomes, proteomes, and metabolic needs (5 – 8). *T. brucei* proliferates as the slender bloodstream form (BSF) in the blood, lymph, skin, adipose tissue, and cerebrospinal fluid (9 – 12). Following a quorum sensing event, slender BSF differentiates from the quiescent, non-dividing stumpy BSF (13). Stumpy BSF parasites are pre-adapted for differentiation and survival in the tsetse fly vector (14 – 16). When a tsetse fly takes an infected blood meal, slender and stumpy BSF are ingested, providing environmental triggers that signal for differentiation to the insect procyclic form (PCF) in the tsetse fly midgut. While slender BSF parasites have the capacity to differentiate from the proliferative PCF, stumpy BSF parasites achieve differentiation more efficiently (14, 15).

The mammalian slender BSF and the insect PCF thrive in nutritionally contrasting environments and produce most of their ATP by different metabolic pathways. While slender BSF parasites may have varying metabolic states depending on the host tissue they inhabit (10, 11), slender BSF parasites in the blood rely heavily on blood glucose to fuel glycolysis (5, 17 – 19). Slender BSF parasites do not express respiratory complexes II (succinate dehydrogenase), III (cytochrome *b*-*c*1 reductase), or IV (cytochrome oxidase) (6, 20) and, therefore, do not have a complete electron transport chain (ETC) or rely on oxidative phosphorylation in the mitochondrion to generate ATP. Because the tsetse fly midgut is not a reliable source of glucose, when BSF parasites differentiate into PCF, they must express and assemble the complete ETC to generate ATP. Similar to other eukaryotes, several subunits of the *T. brucei* ETC are encoded in the mitochondrial genome (21, 22). However, unlike other eukaryotes, *T. brucei* and other kinetoplastid protozoans must post-transcriptionally modify some mitochondrial mRNAs to generate translatable open reading frames (ORFs) by a mechanism called uridine insertion/deletion (U-indel) editing (23, 24).

U-indel editing is a coordinated process that requires the participation of the RNA editing catalytic complexes, the non-catalytic RNA editing substrate-binding complex (RESC), and several accessory factors (24). The precise number and position of uridine insertions and deletions are informed by *trans*-acting guide RNAs (gRNAs) that are also encoded in the mitochondrial genome (25, 26). The only exception is cytochrome oxidase subunit II (COII) mRNA, a minimally edited mRNA whose own 3′UTR acts as a *cis*-guide to direct four uridine insertions to correct its own ORF (27, 28). Additional edited mRNAs include the moderately edited CYb and MURF2 that only require two gRNAs to complete a small editing domain (29), and the nine pan-edited mRNAs that require dozens of gRNAs to extensively edit almost the entire length of the mRNAs. The mitochondrial mRNAs that require U-indel editing encode subunits of the mitochondrial ribosome and respiratory complexes I (NADH dehydrogenase), III, IV, and V (ATP synthase). Although the BSF mitochondrion does not perform oxidative phosphorylation, complex V is still essential in BSF as it runs in reverse and hydrolyzes ATP to help maintain mitochondrial membrane potential (30, 31). Therefore, U-indel editing is required for parasite survival (32, 33).

While U-indel editing is essential, not all mitochondrial mRNAs are constitutively edited throughout the *T. brucei* life cycle. Previous studies on the developmental regulation of U-indel editing often relied on less quantitative techniques (34 – 39) or compared monomorphic BSF parasites that have lost their ability to differentiate into stumpy BSF to axenically cultured PCF (40 – 42). Changes in the abundances of total or edited mRNAs have not been previously quantified in pleomorphic *T. brucei* parasites as they progressed from slender BSF to PCF. Therefore, there has been no resolution of how and when U-indel editing changes during the transition between slender BSF and PCF or which mRNAs are regulated in a pleomorphic strain. Thus, we aimed to address several unanswered questions about the developmental regulation of U-indel editing during the BSF-to-PCF transition in a pleomorphic *in vitro* culture model. We wanted to know (i) which mRNAs are regulated at the level of either mRNA abundance or mRNA editing as *T. brucei* progressively transitions through slender BSF, stumpy BSF, and PCF; (ii) at which point during the BSF-to-PCF transition do these changes occur; (iii) what stimulates the developmental changes in mRNA abundance and/or editing; and (iv) do the regulated mRNAs respond to the same stimuli. Here, we show that the mitochondrial-encoded cytochrome mRNAs (COI, COII, COIII, and CYb) are rapidly upregulated only after stumpy BSF is induced to differentiate to PCF, the upregulation of distinct cytochrome mRNAs is achieved by different mechanisms, and the upregulation of COII mRNA editing is uniquely temperature-responsive and augmented by the depletion of the RDK1 kinase that represses BSF-to-PCF differentiation.

## RESULTS

### The mitochondrial-encoded cytochrome mRNAs are upregulated early in the transition from BSF to PCF

**Fig 1:**
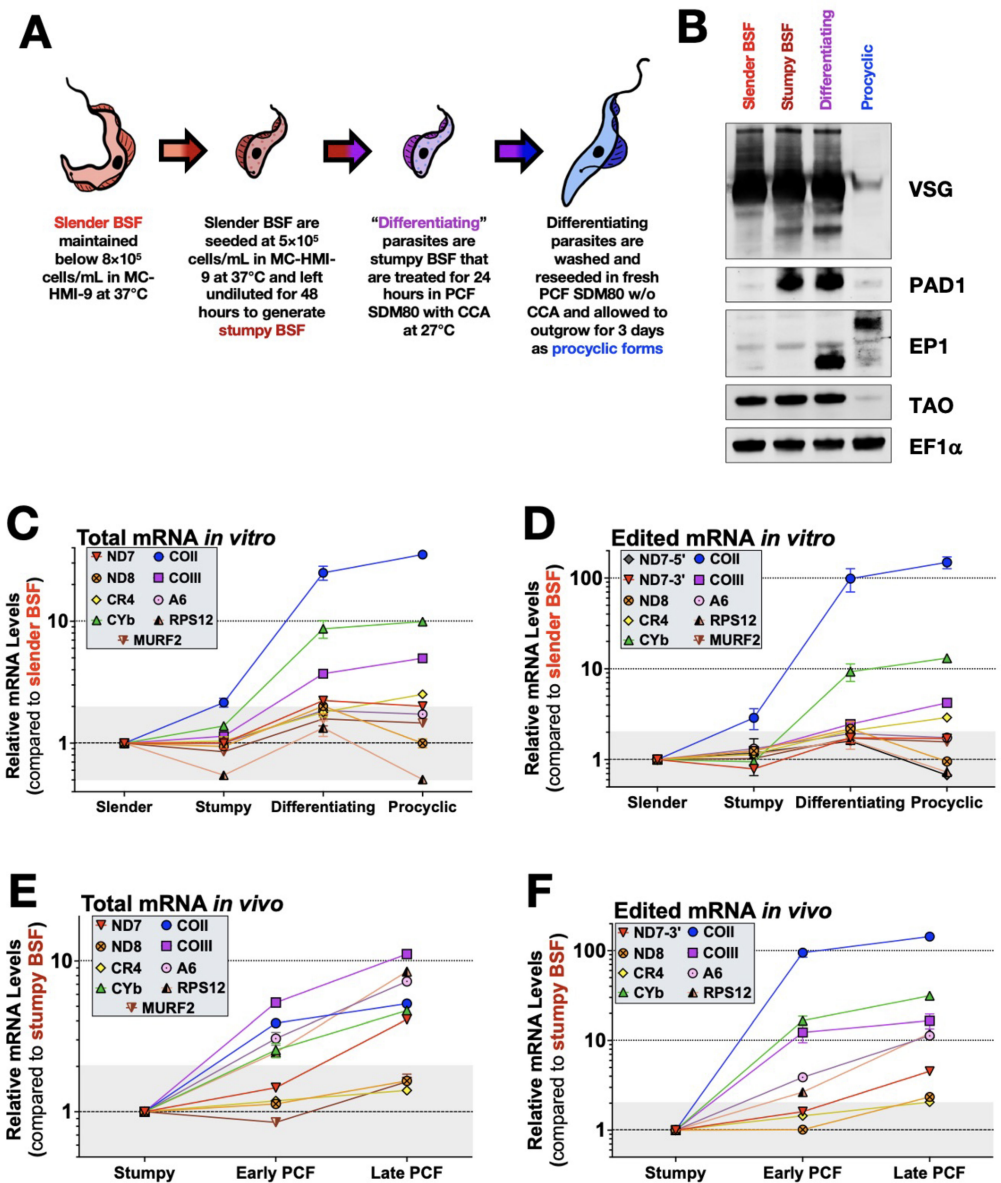
The abundance and/or editing of mitochondrial cytochrome mRNAs is upregulated when stumpy BSF is triggered to differentiate to PCF both *in vitro* and *in vivo*. (**A**) Schematic illustrating how *T. brucei* parasites were cultured and treated during the *in vitro* slender BSF-to-PCF differentiation workflow. Parasites are not drawn to scale. (**B**) Western blot of total cell lysates from slender BSF, stumpy BSF, differentiating, and PCF parasites to show expression of life cycle stage markers for VSG, PAD1, EP1 procyclin, and TAO. EF1α was used as a loading control. Quantitative RT-PCR analysis of (**C**) total and (**D**) edited mitochondrial mRNAs for *in vitro* cultured parasites differentiating from slender BSF to PCF. Quantitative RT-PCR analysis of **(E**) total and (**F**) edited mitochondrial mRNAs for *in vivo* cultured parasites differentiating from stumpy BSF to early and late PCF in teneral tsetse flies.

First, we aimed to determine the timing at which the abundance and editing of mitochondrial mRNAs are regulated at different stages during the slender BSF-to-PCF transition. To do this, we used the pleomorphic EATRO1125 AnTat1.1 90-13 cell line that retains the abilities to grow reliably as slender BSF, to differentiate into stumpy BSF in a cell density-dependent manner, and to readily differentiate into PCF *in vitro* (43). We isolated RNA and cell lysate samples from triplicate *T. brucei* cultures as they progressed in the life cycle from slender BSF to PCF and confirmed the protein expression of stage-specific markers by Western blot (Fig. 1A and B). As expected, slender and stumpy BSF parasites express variable surface glycoprotein (VSG). Stumpy BSF parasites exposed to citrate/*cis*-aconitate (CCA) in PCF SDM80 medium at 27°C for 24 h (labeled as “differentiating”) retain VSG expression, while the VSG signal is lost in fully differentiated PCF parasites. The stumpy BSF marker, PAD1, is only detectable in stumpy BSF and differentiating parasites. We also detected EP1 procyclin expression in the differentiating and PCF parasites, but we observed that procyclin migrates as a higher band in PCF likely due to glycosylation (44). These data confirm the identity of the life cycle stages harvested for RNA isolation. This is particularly important for confirming that the differentiating parasites are intermediate forms between BSF and PCF as VSG, PAD1, and TAO are still detectable while procyclins begin their expression in their unglycosylated forms.

Next, we determined the relative total abundances of COII, CYb, MURF2, and six pan-edited mRNAs (ND7, ND8, CR4, COIII, A6, and RPS12) as the parasites transitioned from slender BSF to PCF using qRT-PCR (29, 41, 45). For COII, CYb, MURF2, and ND7, primers were designed to target never-edited regions within the respective mRNAs (29, 41). For the remaining pan-edited mRNAs, total qPCR primers could not be designed to target only never-edited regions due to the extensive editing of these mRNAs. Therefore, primers were designed to target the 5′ never-edited region and an extreme 5′ pre-edited sequence within the editing domain as previously described to detect the majority of the mRNA population, referred to herein as “total” (41, 45). The relative total abundances of ND7, ND8, CR4, A6, RPS12, and MURF2 mRNAs followed a similar pattern where abundance is unchanged in stumpy BSF and modestly increased (1.3- to 2.2-fold) in differentiating parasites (Fig. 1C). These mRNAs generally maintained the same relative abundance in PCF as in differentiating parasites, except for ND8 and RPS12, which returned to levels comparable to, or slightly below, those in slender BSF (Fig. 1C). By contrast, total COII, COIII, and CYb mRNAs exhibited a distinct pattern in which their abundances significantly increased in differentiating parasites (3.7- to 24.9-fold) and continued to increase in PCF parasites (5.0- to 35.2-fold) relative to slender BSF. COII mRNA was the only mRNA to increase abundance (2.2-fold) in stumpy BSF, and it continued to exhibit the most striking increase of 35.2-fold from slender BSF to PCF (Fig. 1C). Most of the increases for COII, CYb, and COIII mRNAs occurred within the first 24 h in differentiating parasites that have not yet fully completed PCF differentiation (Fig. 1B). In fact, cytochrome mRNA upregulation occurred within 6 h (Fig. S1). We also determined the relative abundances of the ND4 and COI mRNAs, which are never-edited mitochondrial mRNAs that encode for subunits for Complex I and IV, respectively. ND4 displayed an mRNA expression pattern similar to ND7 and ND8, whereas COI mRNA abundance was increased 6.7- and 7.0-fold in differentiating and PCF parasites, respectively (Fig. S2A). Thus, total abundances of mRNAs encoding subunits of respiratory cytochrome complexes III and IV are specifically and rapidly upregulated in response to the differentiation trigger of 27°C and CCA.

Next, we determined the relative levels of the edited versions of the mitochondrial mRNAs. Edited ND8, A6, RPS12, and MURF2 mRNAs displayed a similar pattern to their total mRNA abundance with little overall change from slender BSF to PCF (Fig. 1D). ND7 mRNA has two distinct editing domains (46), which, in PCF, were differentially regulated compared to BSF. While the 3′ edited domain (ND7-3′) maintained a similar pattern as overall ND7 mRNA abundance, modestly increasing twofold in PCF, the 5′ edited domain (ND7-5′) decreased 2.8-fold in fully differentiated PCF parasites (Fig. 1D; Fig. S2B). Edited CR4 and COIII mRNA levels were increased 2.9- and 4.2-fold, respectively, in PCF parasites. In contrast to these modest changes, the relative increases of edited CYb and COII mRNAs were 13.1- and 148.6-fold, respectively, in PCF compared to slender BSF (Fig. 1D), with most of the changes occurring within the first 6 h of differentiation (Fig. S1). Thus far, the data reveal that the abundance of both total and edited mitochondrial cytochrome mRNAs is upregulated during BSF-to-PCF differentiation in *in vitro* culture, with COII exhibiting the earliest and most drastic changes (Fig. 1C and D).

To determine if the patterns of regulation observed *in vitro* are reflected during an *in vivo* tsetse fly infection, we infected teneral flies with stumpy BSF EATRO1125. Infected tsetse flies were dissected up to 7 days post infection. RNA was isolated from infected midguts, and fold changes of total and edited mRNAs of tsetse fly parasites relative to stumpy BSF were measured by qRT-PCR. When comparing day 3 midgut (early PCF) and day 7 midgut (late PCF) parasites (7, 44) to stumpy BSF, we found that the total abundance of all tested mitochondrial mRNAs except ND7, ND8, and CR4 steadily increased from stumpy BSF to late PCF (Fig. 1E). This contrasts with *in vitro* measurements, where only the cytochrome mRNAs substantially increased in abundance in PCF (Fig. 1C). Among the cytochrome mRNAs, COIII mRNA abundance increased the most during *in vivo* infections while it had the lowest increase *in vitro* (Fig. 1C and E). When we compared the relative abundance of edited mRNAs during *in vivo* tsetse fly infections (Fig. 1F), we found that, as observed *in vitro*, edited cytochrome mRNAs were the most upregulated, especially COII mRNA with a 143.7-fold increase in late PCF compared to stumpy BSF. We also note that edited A6 and RPS12 mRNAs exhibited greater increases *in vivo* than *in vitro* (Fig. 1F). Overall, these data demonstrate that the editing and abundance of the cytochrome mRNAs are upregulated during the BSF-to-PCF differentiation process both *in vitro* and *in vivo*. Strikingly, not only are the total and edited COII mRNA increases triggered at an earlier time point relative to other mRNAs, but the upregulation of edited COII mRNA in PCF parasites significantly outpaces the increase in total COII mRNA abundance, suggesting a dramatic impact on the COII mRNA editing process during differentiation (Fig. 1C through F).

### The editing efficiency of COII and CYb, but not COIII, mRNAs increases during differentiation from BSF to PCF

**Fig 2:**
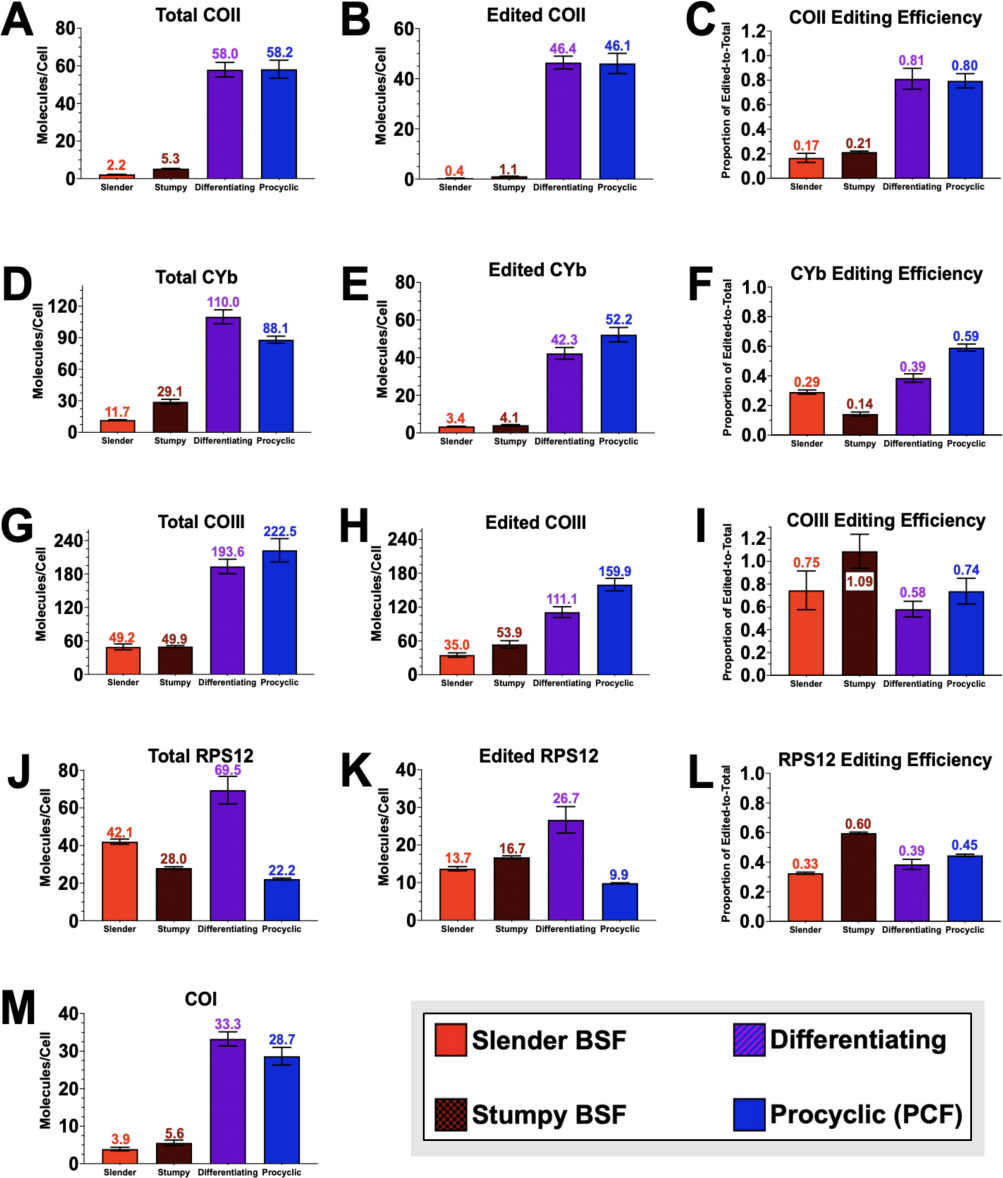
The editing efficiency of COII and CYb mRNAs increases during BSF-to-PCF differentiation. Droplet digital PCR analysis was used to calculate the mRNA copy number per cell of total and edited mRNAs and their corresponding editing efficiency for (**A–C**) COII, (**D–F**) CYb, (**G–I**) COIII, and (**J–L**) RPS12 during *in vitro* slender BSF-to-PCF differentiation. (**M**) The mRNA copy number per cell of COI mRNA was calculated using ddPCR.

While qRT-PCR shows relative changes in mRNA levels between life cycle stages, the absolute abundances of the mitochondrial mRNAs have never been determined. Larger relative fold changes of non-abundant mRNAs may have a different biological implication than smaller fold changes of highly abundant mRNAs. Therefore, knowing absolute copy numbers provides insight into the relative roles of changes in mRNA abundance and editing in the ultimate levels of edited mRNAs. To determine the copy numbers of the developmentally regulated cytochrome mRNAs, we performed droplet digital PCR (ddPCR) (47). We also analyzed RPS12 mRNAs, which were not developmentally upregulated in PCF *in vitro*. Knowing the precise numbers of total and edited mRNAs for a given mRNA allowed us to calculate the ratio of edited mRNA-to-total mRNA to determine “editing efficiency.” This value allows us to determine if the extent of editing for a particular mRNA is controlled solely by its abundance. Total and edited COII mRNAs have a low abundance in BSF (Fig. 2A and B), but COII mRNA has a low editing efficiency that indicates most COII mRNA is not edited in BSF (Fig. 2C). When stumpy BSF is triggered to differentiate to PCF, COII mRNA abundance increases more than 10-fold and the editing efficiency starkly increases (Fig. 2A through C). Total and edited CYb mRNAs also have low abundances in BSF, but unlike COII mRNA, the editing efficiency of CYb mRNA progressively increases from stumpy BSF to PCF (Fig. 2D through F). These data show that while COII and CYb mRNAs are not particularly abundant in BSF parasites, the majority of COII and CYb mRNAs remain mostly pre-edited. This suggests that the editing of COII and CYb mRNAs is actively suppressed in BSF until the differentiation stimulus is received although the mRNAs have different kinetics for achieving peak editing efficiencies.

Similar ddPCR analyses for COIII mRNAs revealed that COIII is the most abundant cytochrome mRNA in BSF (Fig. 2G and H). We found that the majority of COIII mRNA is edited in BSF (Fig. 2I), contrary to results shown in previous studies using monomorphic BSF cell lines that cannot readily differentiate into PCF (48, 49). However, primers to detect edited COIII do not align at the extreme 5′ end region of COIII’s edited domain (50) and so require only partial editing of the mRNA for annealing. Therefore, we redesigned primers to detect edited COIII at its extreme 5′ region to detect almost completely edited COIII mRNA. Compared to the established edited COIII primers, we found that the relative fold changes were comparable (Fig. S2C). Thus, we conclude that the editing efficiency of COIII mRNA is nearly constant during the BSF-to-PCF transition and that COIII mRNA upregulation is driven primarily by total mRNA abundance. We also quantified the numbers of molecules per cell for RPS12 mRNAs, which did not display the same developmental pattern as the cytochrome mRNAs, and the never-edited COI mRNA (Fig. 1C through F). Total and edited RPS12 mRNA abundances oscillate and are lower in abundance in PCF parasites than in slender BSF (Fig. 2J and K). RPS12 editing efficiency also does not follow an obvious pattern, and there is no drastic shift in editing efficiency of RPS12 from BSF to differentiating parasite (Fig. 2L). Lastly, similar to COII and CYb, COI mRNA is not particularly abundant in BSF but prominently increases in differentiating parasites and PCF (Fig. 2M). Altogether, these data establish that both the abundance and editing efficiency of COII and CYb mRNAs are increased in response to the differentiation signal of 27°C and CCA treatment, while COI and COIII mRNAs are primarily regulated at the level of abundance.

### A reduction in temperature specifically stimulates the partial upregulation of COII and COIII mRNA editing

**Fig 3:**
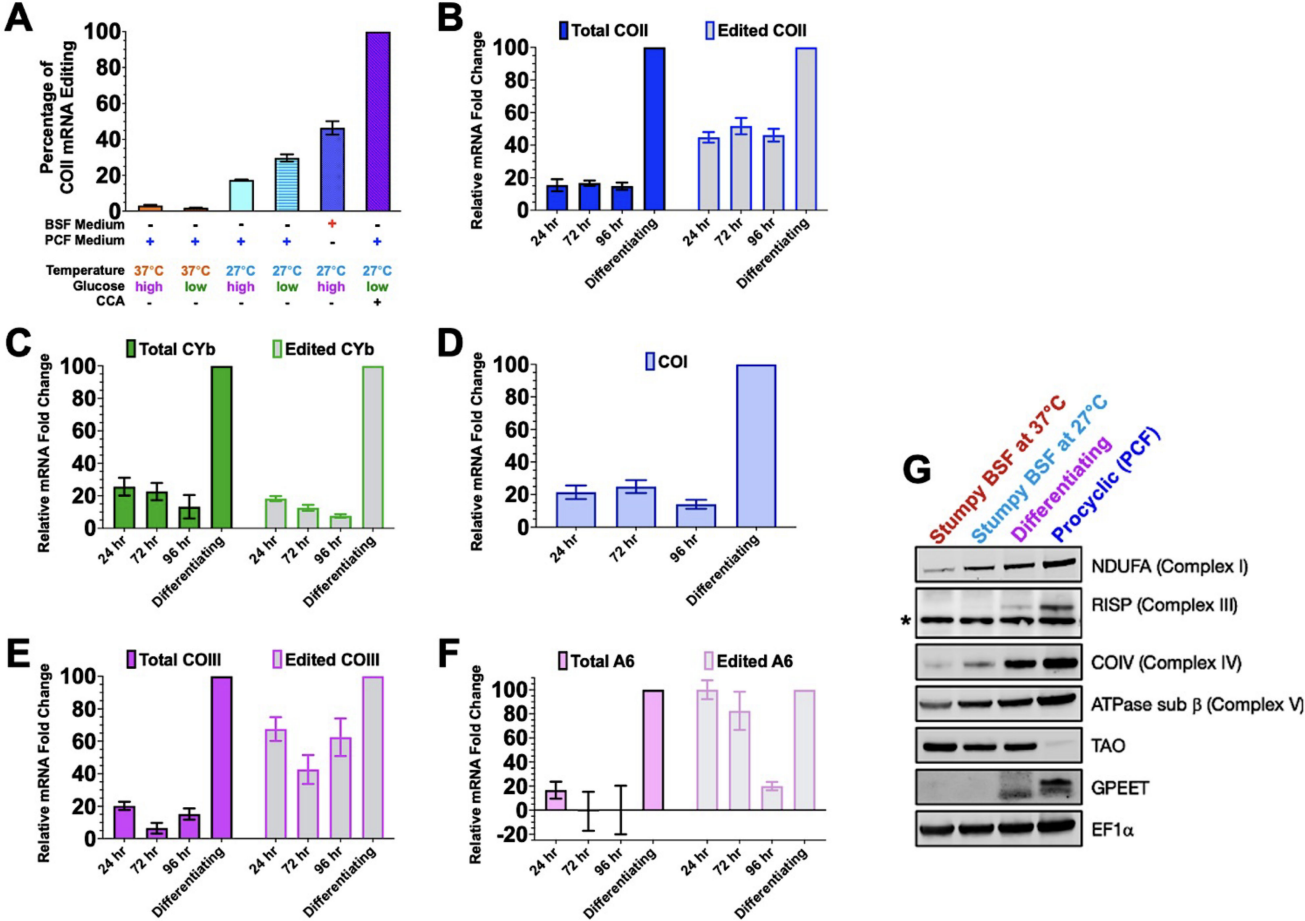
Temperature reduction partially stimulates editing efficiency of COII and COIII mRNAs. (**A**) Quantitative RT-PCR analysis of edited COII mRNA levels in stumpy BSF cultured in different conditions for 24 h (compared to stumpy BSF in standard conditions at 37°C) to dissect the effect of discrete environmental cues. The relative fold change of edited COII mRNA in differentiating parasites compared to stumpy BSF parasites was set to 100%. (**B–F**) Quantitative RT-PCR analysis on COII, CYb, COI, COIII, and A6 mRNA levels in stumpy BSF parasites subjected to prolonged exposure to 27°C. (**G**) Western blot analysis of select nuclear-encoded respiratory subunits (NDUFA, RISP, COIV, ATPase sub β), TAO, and GPEET procyclin. EF1α is a loading control. The asterisk (*) indicates a non-specific band.

We next wanted to determine which discrete differentiation signals account for the dramatic upregulation of edited COII mRNA differentiating parasites (Fig. 2). Edited COII mRNA levels in differentiating parasites (stumpy BSF that was cultured in glucose-deficient PCF SDM80 medium at 27°C in the presence of CCA for 24 h) were referenced as 100% (Fig. 3A). We began by asking whether the change in temperature or glucose concentration mediated this effect, as both have been implicated as differentiation cues for BSF parasites to differentiate to PCF (18, 43, 51). Stumpy BSF was resuspended in PCF medium that was supplemented with either 5.55 mM glucose or 50 mM *N*-acetyl-d-glucosamine (+GlcNAc) to inhibit uptake of residual glucose at 37°C or 27°C for 24 h (Fig. 3A). Edited COII mRNA was not increased when stumpy BSF was kept at 37°C, regardless of the presence or absence of glucose (Fig. 3A); furthermore, these cells did not outgrow as PCF and died 24 h later (Fig. S3A). In contrast, when stumpy BSF parasites were cultured at 27°C, edited COII mRNA levels reached 14.9% and 27.2% of that in differentiating cells in the presence of glucose in the absence of glucose, respectively (Fig. 3A). Thus, temperature is the more important factor in upregulating COII mRNA editing, while glucose concentration has only a minor effect. The upregulation of COII mRNA editing is reflected in the parasite outgrowth as PCF under these conditions (Fig. S3A).

Thus far, stumpy BSF parasites were shifted from the BSF medium to the PCF medium. To determine whether temperature reduction alone is sufficient to upregulate COII mRNA editing, we harvested stumpy BSF parasites and cultured them in BSF medium at 27°C for 24 h to eliminate the contribution of other components of PCF medium. Surprisingly, stumpy BSF parasites cultured in BSF medium at 27°C increased COII mRNA editing even more than parasites cultured in PCF medium at 27°C (Fig. 3A), suggesting that other components of PCF medium stimulate competing signals.

We next asked if the increase of COII mRNA editing is augmented by prolonged 27°C stimulus and whether the abundance and/or editing of other mRNAs are temperature-responsive. We found that prolonged exposure to the cold stimulus did not further increase COII mRNA editing (Fig. 3B). Of the other mRNAs tested, the abundance and/or editing of COI and CYb mRNAs were not upregulated (Fig. 3C and D). Temperature reduction stimulated the editing of COIII mRNA without the concomitant increase in COIII mRNA abundance (Fig. 3E), which was surprising as COIII mRNA abundance increase outpaces editing during *in vitro* differentiation (Fig. 2G and H). Culturing stumpy BSF at 27°C causes edited A6 mRNA levels to increase without affecting overall A6 mRNA abundance, but this increase was transient and could not be maintained (Fig. 3F). Furthermore, while culturing stumpy BSF at 27°C does prolong their life span, they do not re-enter the cell cycle and outgrow as PCF (Fig. S3B). These data demonstrate that temperature is a key stimulus in regulating COII and COIII mRNA editing and that the signals that regulate editing efficiency and mRNA abundance are separable.

### Specific nuclear-encoded respiratory subunits are cold-responsive

Upregulation of nuclear-encoded subunits of the respiratory complexes is a step that is presumably necessary for the assembly of functional ETC complexes. Therefore, we next determined if the protein levels of nuclear-encoded subunits increase in parallel with the upregulation in editing of the mitochondrial mRNAs and whether their abundances are cold-responsive. To this end, we performed Western blot analysis during the BSF-to-PCF transition using antibodies against NDUFA (complex I), Rieske protein/RISP (complex III), COIV (complex IV), and ATPase subunit β (complex V). Stumpy BSF parasites cultured in the BSF medium at 27°C increase NDUFA, COIV, and ATPase subunit b expression but not RISP (Fig. 3G). This was intriguing because it means that COIV expression and COII mRNA editing upregulation (complex IV components) are cold-responsive, while RISP expression and CYb mRNA editing (complex III components) are not. Furthermore, COIV expression was higher in the differentiating parasites that received the full differentiation signal than stumpy BSF parasites cultured at 27°C (Fig. 3G) similar to the upregulation of COII mRNA editing. In the differentiating parasites, RISP is detectable by Western blot but does not reach maximal steady-state levels until differentiation to PCF is complete (Fig. 3G) similar to the progressive increase in editing efficiency of CYb mRNA (Fig. 2F). Moreover, stumpy BSF parasites that were cultured at 27°C did not begin expression of GPEET procyclin protein, while differentiating parasites have detectable, unglycosylated GPEET (Fig. 3G). This further supports that, while temperature reduction is a necessary environmental cue to begin COIV expression and COII mRNA editing upregulation, it is not sufficient to trigger full differentiation to PCF.

### Slender BSF upregulates COII mRNA editing in response to cold stimulus

**Fig 4:**
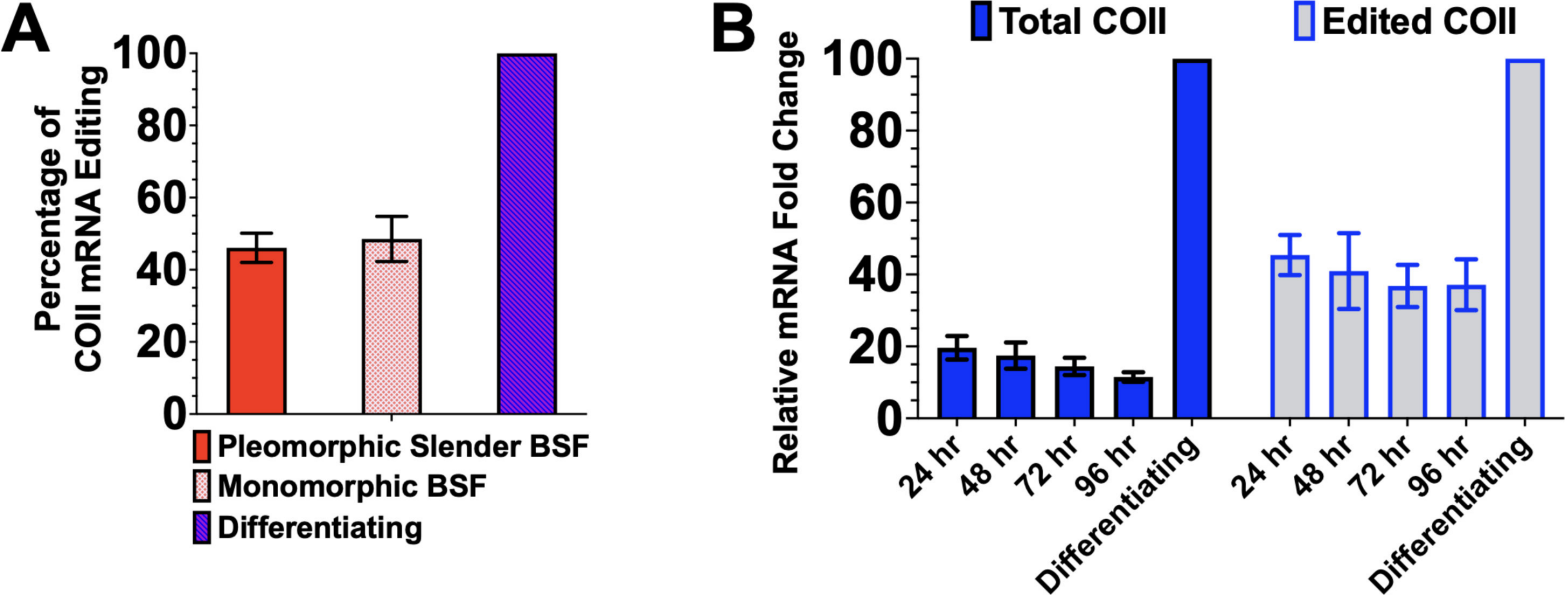
The cold-responsive upregulation of COII mRNA editing is an inherent trait of slender BSF parasites. (**A**) Quantitative RT-PCR analysis of edited COII mRNA in pleomorphic EATRO1125 slender BSF parasites cultured in MC-HMI-9 (solid red bars) or monomorphic Lister 427 BSF parasites cultured in HMI-9 (checkered pink bars) at 27°C for 24 h compared to corresponding BSF parasites cultured at 37°C. The relative fold change of edited COII mRNA in differentiating parasites compared to slender BSF parasites was set to 100%. (**B**) Quantitative RT-PCR analysis of total and edited COII mRNA in pleomorphic slender BSF EATRO1125 parasites cultured in MC-HMI-9 medium at 27°C up to 96 h. The relative fold change of total or edited COII mRNA in differentiating parasites compared to slender BSF parasites was set to 100%.

Stumpy BSF exhibits various molecular pre-adaptations for differentiation to PCF compared to slender BSF (52). To determine if upregulation of COII mRNA editing in response to a cold stimulus requires stumpy BSF adaptations, we cultured slender BSF EATRO1125 parasites in BSF medium for 24 h at 27°C and measured edited COII mRNA. Compared to slender BSF at 37°C, we observe an upregulation of edited COII mRNA in slender BSF at 27°C (46.1% of the increase observed in differentiating parasites) (Fig. 4A). To determine if COII mRNA editing upregulation requires the full ability to differentiate to PCF, we performed a similar experiment with monomorphic Lister 427 BSF parasites, and we observed an increase in edited COII mRNA comparable to that in pleomorphic slender BSF parasites (Fig. 4A). To determine if the upregulation of edited COII mRNA could be maintained in slender BSF, as it is in stumpy BSF, or if this increase was more transient, we cultured slender BSF at 27°C for 96 h and performed qRT-PCR analysis to evaluate the levels of total and edited COII mRNA. The increase in total and edited COII mRNA remained elevated in slender BSF during prolonged exposure to 27°C (Fig. 4B). Altogether, these data show that the factors to stimulate and maintain the cold-responsive upregulation of COII mRNA editing are present in both slender and stumpy BSFs.

### RDK1 depletion couples with the cold-responsive signaling pathway to further upregulate COII mRNA editing

**Fig 5:**
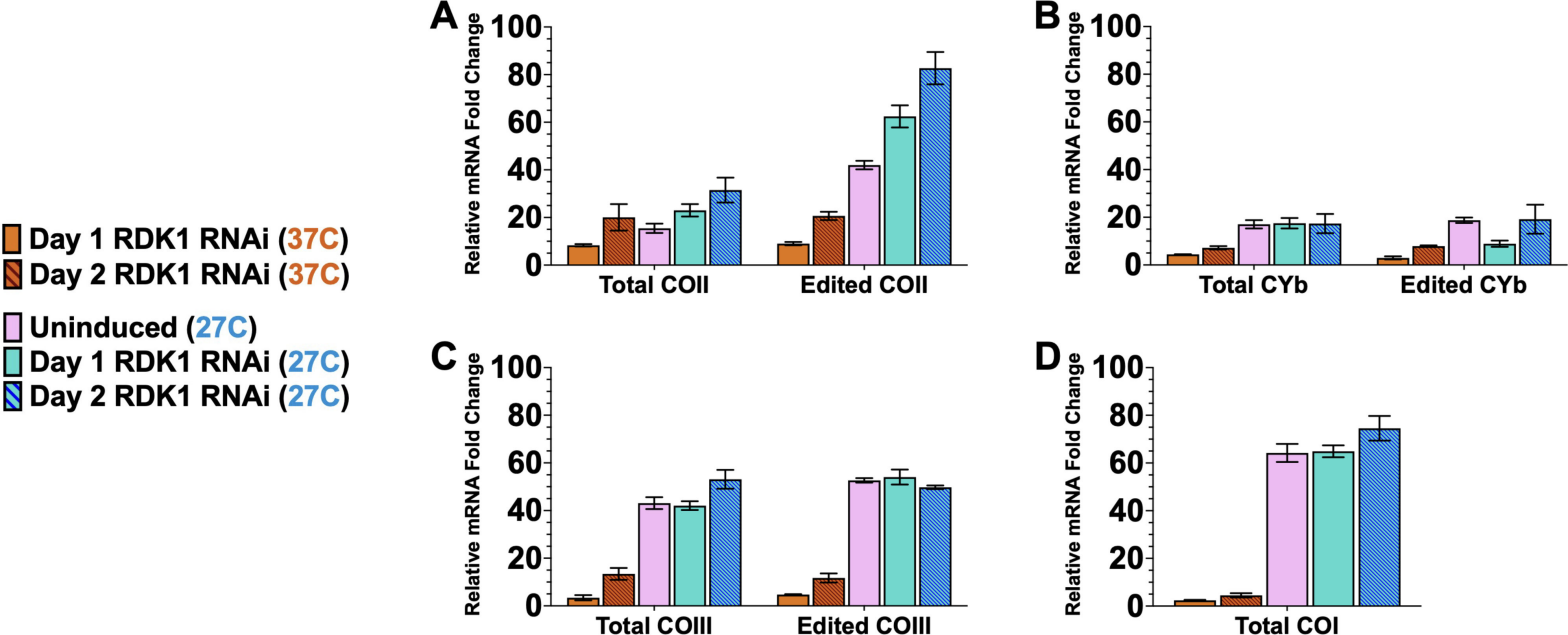
RDK1 depletion couples with temperature reduction to stimulate COII mRNA editing. (**A–D**) Quantitative RT-PCR analysis of total/edited (**A**) COII, (**B**) CYb, (**C**) COIII, and (**D**) COI mRNA in BSF RDK1 RNAi parasites cultured in MC-HMI-9. RDK1 depletion was induced by with treatment of doxycycline (0.5 µg/mL) for up to 2 days either at 37°C or at 27°C. Uninduced BSF RDK1 RNAi cells exposed to 27°C for 24 h are also shown (pink bars). The relative mRNA fold changes in procyclic parasites compared to uninduced BSF RDK1 RNAi parasites cultured at 37°C were set to 100%.

**Fig 6:**
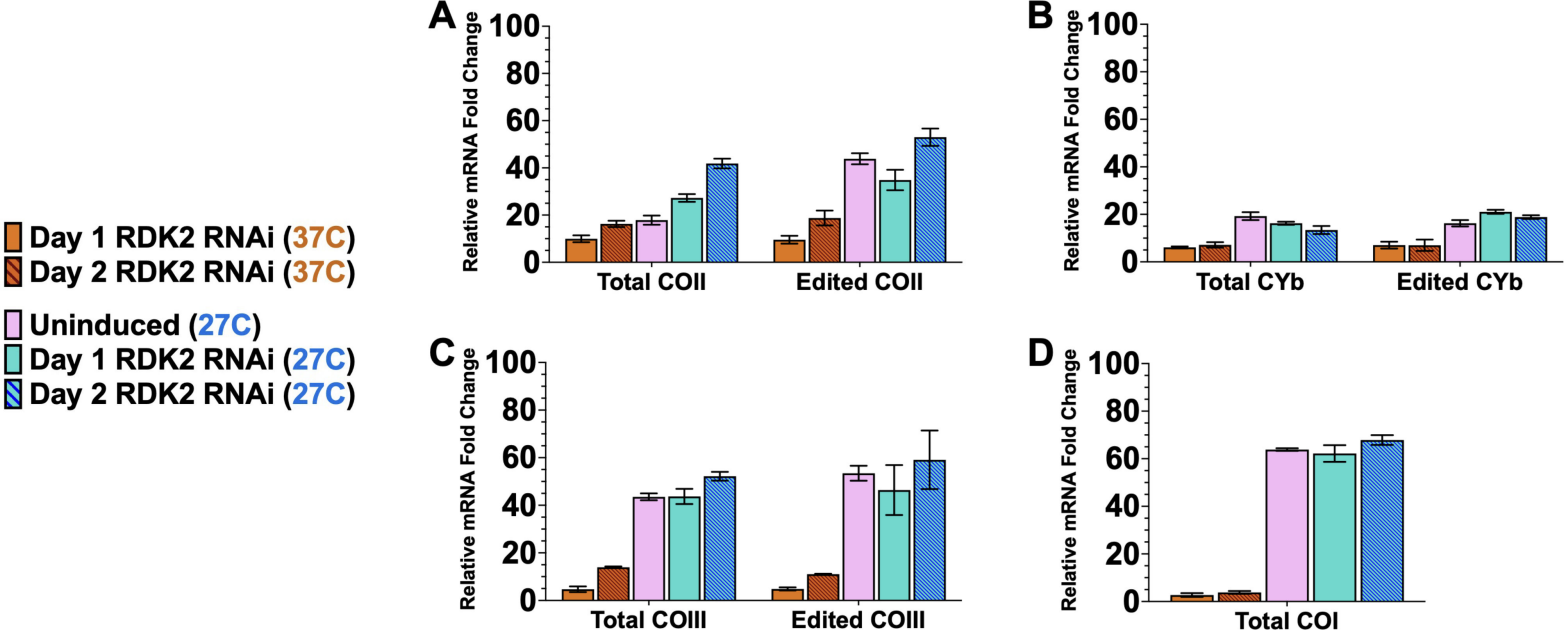
RDK2 depletion does not stimulate COII mRNA editing. (**A–D**) Quantitative RT-PCR analysis of total/edited (**A**) COII, (**B**) CYb, (**C**) COIII, and (**D**) COI mRNA in BSF RDK2 RNAi parasites cultured in MC-HMI-9. RDK2 depletion was induced by with treatment of doxycycline (0.5 µg/mL) for up to 2 days either at 37°C or at 27°C. Uninduced BSF RDK2 RNAi cells exposed to 27°C for 24 h are also shown (pink bars). The relative mRNA fold changes in procyclic parasites compared to uninduced BSF RDK2 RNAi parasites cultured at 37°C were set to 100%.

We demonstrated that temperature reduction is a critical stimulus in promoting the editing efficiency of some cytochrome mRNAs. However, previous studies identified two kinases that, when depleted in BSF, can trigger differentiation to PCF at 37°C (53). The repressors of differentiation kinase 1 (RDK1) and 2 (RDK2) are expressed in BSF to prevent the parasites from spontaneously differentiating into PCF. Depletion of RDK1 causes transcriptome remodeling, including the downregulation of VSG mRNA and the upregulation of procyclins and some nuclear-encoded cytochrome mRNAs. Depletion of RDK2 robustly induces procyclin expression. However, the effects of RDK1 or RDK2 depletion on mitochondrial cytochrome mRNAs were not studied. Therefore, we evaluated the effect of RDK1 or RDK2 depletion on the abundance and editing of the mitochondrial cytochrome mRNAs at 37°C and 27°C. We generated doxycycline-inducible RNAi cell lines that depleted either RDK1 or RDK2 in slender BSF parasites (Fig. S4) (53). We first cultured BSF RDK1 RNAi parasites in either the absence or presence of doxycycline for up to 48 h at 37°C and 27°C to induce RDK1 depletion and measured the change in the abundance of total and edited COII, CYb, COIII, and COI mRNAs, setting the fold change in PCF parasites relative to slender BSF parasites cultured at 37°C at 100%. For COII mRNA, we observed that RDK1 depletion at 37°C did not cause a substantial increase in the levels of total or edited mRNAs, both of which remained below 20% of PCF levels (Fig. 5A). Uninduced slender BSF RDK1 RNAi parasites cultured at 27°C for 24 h exhibited a partial stimulation of COII mRNA editing, as previously observed (Fig. 4A). Remarkably, depleting RDK1 at 27°C caused an even greater increase in the levels of edited COII mRNA, which reached 82.7% of PCF levels (Fig. 5A). Regarding CYb mRNA, depleting RDK1 neither at 37°C nor at 27°C stimulated the increase in total or edited mRNA (Fig. 5B). For COIII mRNA, while exposure to 27°C culture conditions does partially stimulate the increase in COIII mRNA abundance, RDK1 depletion did not further increase COIII mRNA abundance or editing (Fig. 5C). Similarly, COI mRNA abundance was partially stimulated by temperature reduction to 27°C but was not responsive to RDK1 depletion (Fig. 5). When examining the effects of RDK2 depletion, we observed no substantial increase in the abundance of edited COII mRNA when cultured either at 27°C or 37°C (Fig. 6A). However, total COII mRNA abundance moderately increased twofold upon RDK2 depletion (Fig. 6A). Similar to RDK1 depletion, RDK2 depletion had little to no effect on the levels of total or edited CYb mRNAs (Fig. 6B), total or edited COIII mRNAs (Fig. 6C), or total COI mRNA (Fig. 6D). From these data, we conclude that, with respect to mitochondrial-encoded cytochrome mRNAs, RDK1 and RDK2 specifically affect COII mRNA. RDK2 contributes to the repression of COII mRNA abundance in BSF. RDK1, along with higher temperatures, contributes to the repression of COII mRNA editing.

### COII mRNA associates with RESC machinery in differentiating PCF parasites

**Fig 7:**
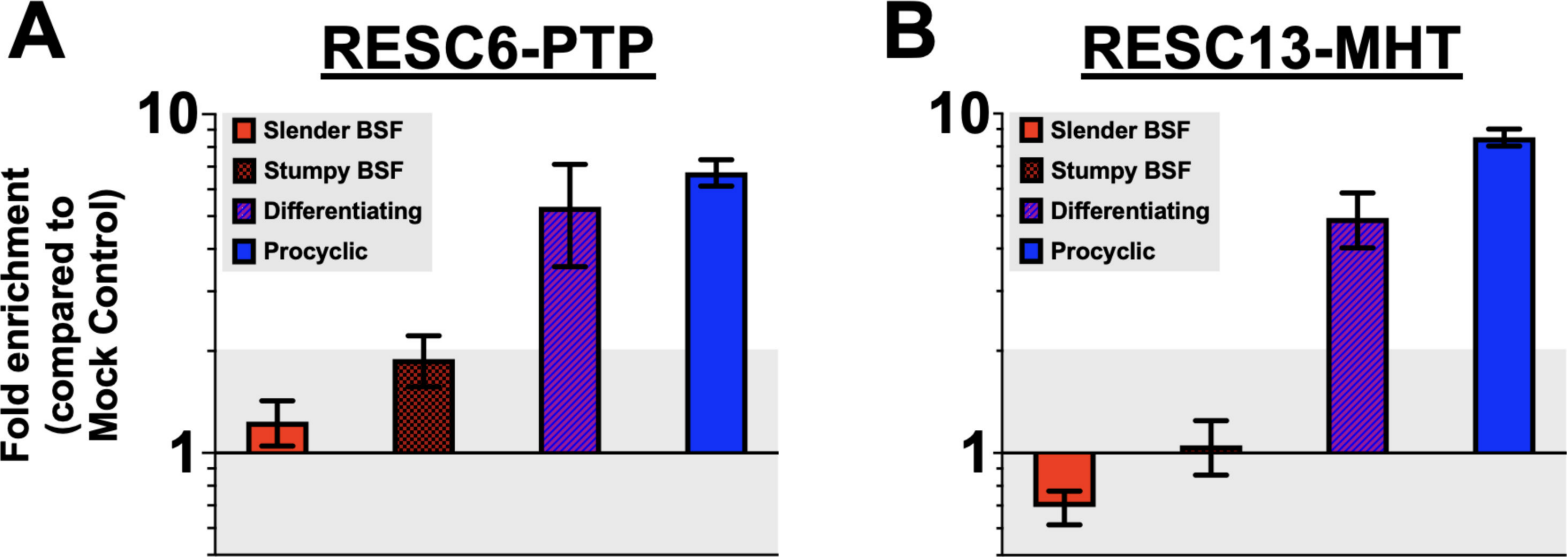
COII mRNA associates with RESC machinery during BSF-to-PCF differentiation. RNA immunoprecipitation assays followed by qRT-PCR (RIP-qPCR) were performed in slender BSF, stumpy BSF, differentiating, and procyclic *T. brucei* parasites expressing either (**A**) RESC6-PTP or (**B**) RESC13-MHT as the protein bait. RESC6-PTP or RESC13-MHT were pulled down by incubating with IgG sepharose beads with clarified parasite lysates. Superdex-200 was used a mock pulldown control. Fold enrichment of total associated COII mRNA was calculated by comparing the COII mRNA signal from IgG pulldowns vs mock control using the ΔΔCt method. 18S rRNA was used as an internal control.

Among the mitochondrial mRNAs that require U-indel editing, COII mRNA is unique because its editing does not utilize a *trans*-acting gRNA. Instead, its own 3′ UTR guides the required four uridine insertions (27, 28). Thus, COII mRNA editing is independent of the gRNA-stabilizing components of RESC, RESC1, and RESC2 (54). Nevertheless, COII mRNA editing is equally as responsive to depletion of the RESC10 protein as are pan-edited mRNAs whose editing requires multiple *trans*-acting gRNAs (55), indicating that COII mRNA editing requires association with at least some RESC modules. To begin to understand the mechanism of COII mRNA editing upregulation, we asked whether its association with the distinct RESC modules changes during differentiation. We generated tagged cell lines in slender BSF 90-13 parasites for RESC6, a component of the guide RNA-binding complex that contains RESC1/2, and RESC13, a component of the RNA editing mediator complexes (23, 24). BSF cell lines harboring chromosomally tagged RESC6-PTP and RESC13-MHT were differentiated *in vitro* to acquire the corresponding PCF cell line. We performed RNA immunoprecipitation (RIP) of RESC6-PTP and RESC13-MHT in slender BSF, stumpy BSF, differentiating, and PCF parasites to quantify the relative association of COII mRNA with RESC during the transition from slender BSF to PCF. In slender BSF, we observed no significant enrichment of COII mRNA with either RESC6 or RESC13 compared to the mock control (Fig. 7A and B). While there may be a slight association of COII mRNA with RESC6 in stumpy BSF (Fig. 7A), no significant enrichment was observed with RESC13 in stumpy BSF (Fig. 7B). However, upon differentiation, we observed a 4.9- to 5.3-fold enrichment of COII mRNA association with the RESC machinery in differentiating parasites that increases to 6.7- to 8.5-fold enrichment in PCF (Fig. 7A and B). Thus, COII mRNA does not notably associate with RESC in BSF but becomes strongly associated following the differentiation trigger. Given the timing and the degree COII mRNA-RESC association, enrichment during differentiation could be due to either the increase in COII mRNA abundance observed during differentiation (Fig. 1C and 2A) or active recruitment to RESC.

### The accessory factor p22 is necessary for the cold-responsive upregulation of COII mRNA editing

**Fig 8:**
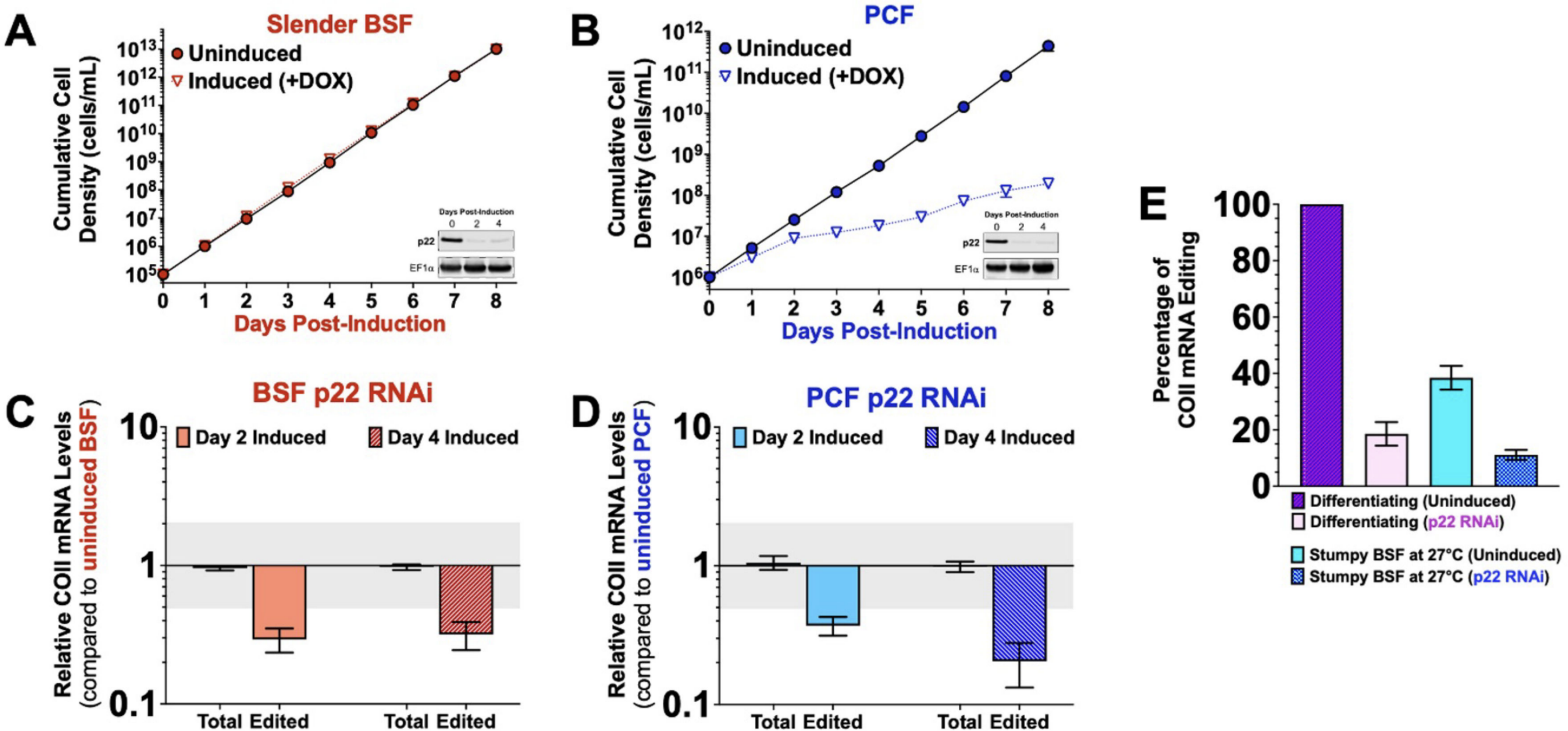
The accessory factor p22 is necessary for the cold-responsive stimulation of COII mRNA editing. (**A–B**) The effect of p22 depletion on the cell growth of (**A**) slender BSF and (**B**) PCF *T. brucei* parasites. The p22 RNAi parasites were grown in their respective standard culture conditions in the absence (uninduced) or presence (induced +DOX) of 0.5 µg/mL doxycycline for up to 8 days. The cumulative cell densities are plotted. The insets show Western blots of total cell lysates from uninduced and induced parasites to confirm p22 knockdown using an antibody specific for p22. EF1a was used as a loading control. Quantitative RT-PCR analysis of total and edited COII mRNA levels in (**C**) slender BSF and (**D**) PCF p22 RNAi cells induced with doxycycline for 2 and 4 days compared to their respective uninduced cells. (**E**) Quantitative RT-PCR analysis demonstrating the effect of p22 depletion on the upregulation of COII mRNA editing in either differentiating parasites or stumpy BSF exposed to 27°C for 24 h.

The editing accessory factor, p22, is a homo-trimer homologous to the multifunctional human p32 protein (56, 57). We previously showed that when p22 is depleted by RNAi, it specifically affects the editing of COII mRNA in PCF (56). To determine if p22 is similarly essential for COII mRNA editing in the pleomorphic strain used in this study, we generated a doxycycline-inducible p22 RNAi cell line in BSF and differentiated BSF transfectants to acquire the corresponding PCF cell line (Fig. 8). We found that p22 depletion reduced the levels of edited COII mRNA in both PCF (Fig. 8C) and BSF (Fig. 8D) without affecting the total COII mRNA abundance. Despite being critical for COII mRNA editing in both life cycle stages, p22 depletion was essential for cell growth only in PCF (Fig. 8A), while p22 depletion had no effect on cell growth in BSF (Fig. 8B), likely due to the dispensability of cytochromes in BSF and the almost complete dependence of PCF on cytochromes. To determine if p22 is required for the upregulation of COII mRNA editing during differentiation or in response to temperature reduction, slender BSF was induced to deplete p22 for 48 h and allowed to differentiate to stumpy BSF. Uninduced and induced (p22-depleted) stumpy BSF parasites were then recultured either in PCF SDM80 medium with CCA at 27°C to induce differentiation or in BSF medium at 27°C for 24 h. qRT-PCR analysis showed that upregulation of COII mRNA editing was impaired during differentiation (Fig. 8E, compare purple vs pink bars) or in response to temperature reduction alone when p22 was depleted (Fig. 8E, compare light blue vs dark blue bars). Thus, we conclude that p22 is necessary for any induction of COII mRNA editing in BSF or PCF.

## DISCUSSION

In the present study, we investigated the developmental dynamics of mitochondrial mRNA abundance and editing in a pleomorphic *T. brucei* strain during differentiation from the mammalian BSF to the insect PCF. Most previous studies used monomorphic BSF parasites that lost their ability to differentiate into stumpy BSF and compared them to axenically cultured PCF, both of which had been separately cultured as BSF and PCF for decades. This continuous axenic culture practice inherently selects against pleomorphic, transmission-competent BSF parasites that can establish infection in tsetse flies as PCF (58). Therefore, investigating the developmental dynamics of mitochondrial U-indel mRNA editing in a system that positively selects for pleomorphic, transmission-competent *T. brucei* parasites was necessary for determining the nature and timing of mitochondrial gene expression in stumpy BSF and intermediary differentiating parasites and to avoid gRNA loss that could be tolerated in one life cycle stage but not another. We also further dissected the influence of different environmental and biochemical triggers during differentiation on mitochondrial gene expression. We show that mainly the cytochrome mRNAs (COI, COII, COIII, and CYb) are developmentally regulated, with changes occurring after stumpy BSF is stimulated to differentiate to PCF. Interestingly, the cytochrome mRNAs do not respond to the same environmental or signaling cues and have different kinetics and methods of upregulation during BSF-to-PCF differentiation.

While the previously reported increases in the abundance of edited COII and CYb mRNAs in PCF relative to BSF remain unchallenged here (35, 36, 40 – 42), studies on the developmental regulation of edited COIII mRNA offer contrasting results. Those using monomorphic Lister 427 BSF parasites report that edited COIII mRNA is undetectable (40 – 42), while an earlier study reports constitutive editing of COIII mRNA in EATRO164 parasites (37). Herein, we observe that EATRO1125 AnTat1.1 slender BSF parasites edit COIII mRNA, but the abundance of edited COIII mRNA increases in PCF, and this increase was especially dramatic during *in vivo* tsetse fly infections (Fig. 1E and F). Surprisingly, we observed a lack of mitochondrial mRNAs that are specifically downregulated during BSF-to-PCF differentiation. Several previous studies report that the edited mitochondrial ND7, ND8, ND9, and CR4 mRNAs are upregulated in BSF compared to PCF (38, 40, 41, 46, 59, 60). Edited ND7, ND8, and ND9 mRNAs encode components of NADH dehydrogenase (complex I), which has an unclear functional role in BSF parasites, apparently lacking enzymatic activity but remaining assembled (20). Furthermore, knocking out nuclear-encoded subunits of NADH dehydrogenase, NUBM and NUKM, demonstrated that the complex is not essential for BSF viability (20). Therefore, it was unclear why BSF would need to upregulate the abundance of edited ND7, ND8, or ND9 mRNAs. We observed no downregulation of edited ND7, ND8, and CR4 mRNAs as slender BSF progresses to PCF, and in some cases, we measured slight increases in their abundances in PCF compared to BSF (Fig. 1D and F). Whether this phenotype is specific to the EATRO1125 AnTat1.1 strain compared to the monomorphic Lister 427 strain is unclear. Regardless, the lower abundance of edited ND7, ND8, and CR4 mRNA in Lister 427 PCF compared to Lister 427 BSF cells is not due to loss of the necessary gRNAs in PCF, as we have previously shown that edited ND7 and ND8 are detectable in PCF and are upregulated when Lister 427 PCF overexpress the RNA-binding protein RBP6 to induce metacyclogenesis (41).

Another major finding that was unexpected was the timing of edited cytochrome mRNA upregulation, specifically the lack of upregulation of cytochrome mRNA editing in stumpy BSF compared to slender BSF. The stumpy BSF mitochondrion is typically regarded as partially reactivated as a preadaptation for increased survival and differentiation to the PCF in the tsetse fly midgut (52, 61). Several studies highlight the increased expression and activity of mitochondrial metabolic enzymes such as the expression of acetate-succinate CoA transferase (ASCT) and the increased enzymatic activity of complex I in stumpy BSF compared to slender BSF (62, 63), and an older study suggests that oxidative phosphorylation may be partially active in stumpy BSF (61). Thus, we were surprised that we observed little to no upregulation in the abundance of total or edited cytochrome mRNAs in stumpy BSF compared to slender BSF (Fig. 1C and D). While there were some minor abundance changes for total COII and CYb mRNAs, their editing efficiencies remain low in stumpy BSF. Furthermore, Western blot analysis of stumpy BSF lysates did not have detectable signals for the nuclear-encoded cytochrome subunits, RISP and COIV (Fig. 3G). These findings are consistent with report that slender BSF and stumpy BSF deplete glucose from culture medium at similar rates (18), suggesting that glycolysis must remain robust in stumpy BSF because the cytochrome subunits for oxidative phosphorylation are not yet produced. Overall, our data establish that upregulation of neither mitochondrial- nor nuclear-encoded cytochrome subunits occurs in stumpy BSF; rather, this process occurs rapidly during the first few days of stumpy BSF-to-PCF differentiation.

Cytochrome mRNAs differed in their patterns of upregulation and the kinetics by which they occur. The use of ddPCR to measure absolute mRNA copy numbers allowed us to distinguish increases in abundance from increases in editing and to calculate editing efficiencies, a metric of mitochondrial U-indel editing that has not been previously measured. While COII and CYb mRNAs exhibited increased editing efficiencies during differentiation, COII mRNA editing efficiency peaked within 24 h of differentiation, whereas CYb mRNA editing efficiency only peaked in completely differentiated PCF (Fig. 2C and F). One explanation of these differing kinetics is that COII mRNA is relatively simple to edit, as it only requires four uridine insertions that are *cis*-guided by the 3′UTR of COII mRNA. This contrasts with CYb mRNA that requires two *trans*-acting guide RNAs to be coordinated by RESC machinery to achieve full editing across a small editing domain. Lastly, the relatively unchanged editing efficiency of COIII mRNA from stumpy BSF to differentiating or PCF parasites suggests that the main strategy of the parasite is to increase overall COIII mRNA abundance, which then translates to increased edited COIII mRNA levels. This is supported by the notable abundance of over 200 COIII mRNAs per cell in PCF compared to ~50 COIII mRNAs per cell in stumpy BSF (Fig. 2G), which was the most abundant mitochondrial mRNA in stumpy BSF that we measured in this study. Interestingly, the fold change increase of edited COIII mRNA abundance from stumpy BSF to tsetse fly-resident early PCF (day 3) was even higher than that observed in PCF derived from *in vitro* culture, while the fold change increases for edited COII and CYb mRNAs were comparable (Fig. 1D and F). This suggests that there are additional signals present in the tsetse fly midgut needed for COIII mRNA editing upregulation that is missing *in vitro*, but nonetheless, this demonstrates that each cytochrome mRNA is uniquely regulated during differentiation.

The distinct patterns of regulation of cytochrome mRNA regulation prompted us to ask which differentiation stimuli contribute to the changes in total and edited mRNA abundances. Although there have been several studies that elucidate the effects of temperature reduction, glucose deprivation, protease exposure, or mild acid treatment to inducing differentiation from BSF to PCF, there have been no studies that investigated how these factors influence the developmental regulation of U-indel mRNA editing. As each of these signals contributes to BSF-to-PCF differentiation with different efficiencies and kinetics (4), we suspected they may differentially affect upregulation of distinct cytochrome mRNAs. Indeed, we observed that temperature reduction serves as the primary sensitizer for increasing the editing efficiency of COII and COIII mRNAs (Fig. 3A through E). Decreased temperature modestly increases the overall abundance of the cytochrome mRNAs but not total A6 mRNA abundance (Fig. 3B through F). Moreover, we observed an increase in edited COIII mRNA in response to an isolated cold shock stimulus without a corresponding increase in total COIII mRNA abundance (Fig. 3E), which differed from what we observed during differentiation where mRNA abundance slightly outpaces the editing of COIII mRNA (Fig. 2I). Thus, the signals that regulate upregulation of COIII mRNA abundance and editing are distinct and separable. The cold-responsive increase in the editing of COII and COIII mRNA was not observed in CYb mRNA.

We also analyzed the effects of temperature on nuclear-encoded cytochrome components. We found that, like the distinct effects of temperature on CYb and COII mRNAs, temperature reduction differentially stimulates the production of nuclear-encoded cytochrome subunits RISP and COIV. Temperature reduction does not induce stumpy BSF parasites to express RISP but does partially stimulate COIV production. This is intriguing because this reveals that temperature reduction specifically contributes to the reactivation of cytochrome oxidase (complex IV) through COII and COIII mRNA editing efficiency and COIV production but not complex III (CYb and RISP). Identifying the signals that mediate this coordination between mitochondrial and nuclear components of distinct mitochondrial complexes will be of future interest.

Finally, we investigated the effects of the differentiation-repressive kinases, RDK1 and RDK2. Both kinases are expressed in BSF to prevent spontaneous differentiation from BSF to PCF; however, the depletion of RDK1 synergizes with several stimuli to increase the production of EP procyclin, the surface coat of PCF parasites, while the depletion of RDK2 does not (53). We found mRNA-specific effects and enzyme-specific effects. Of the four mRNAs tested, only COII mRNA levels notably changed upon RDK1 or RDK2 depletion. RDK1 depletion couples with temperature reduction to dramatically increase the editing efficiency of COII mRNA. In contrast, RDK2 depletion moderately increases the abundance of COII mRNA but not the editing efficiency. It is not known if depletion of RDK1 or RDK2 inactivates a repressive signal that suppresses the editing of COII mRNA or if substrate specificity changes during differentiation to conduct a stimulatory signal to increase COII mRNA abundance and editing efficiency before RDK1 and RDK2 protein levels decrease. Identification of factors comprising RDK1/2 and cold-responsive signaling pathways, as well as the mechanisms governing the developmental increase in CYb mRNA editing, will be exciting future research directions.

## MATERIALS AND METHODS

For buffer compositions, antibody dilutions and sources, and primer nucleotide sequences not detailed here, please refer to Text S1 in the supplementary material.

### *T. brucei* cell culture and maintenance

In this study, we used the pleomorphic EATRO1125 AnTat1.1 90–13 *T. brucei* cell line (43). For *in vitro* culture, slender bloodstream form (BSF) parasites were cultured at 37°C with 5% CO_2_ in modified HMI-9 medium supplemented with 10% heat-inactivated fetal bovine serum (FBS), 1.1% methylcellulose, 2.5 µg/mL G418, and 2.5 µg/mL hygromycin B (64, 65). Slender BSF parasites were maintained below 1 × 10^6^ cells/mL and subcultured every 1–2 days with fresh modified HMI-9 to prevent cell density-dependent differentiation to the non-dividing stumpy BSF (4, 13, 66). Monomorphic Lister 427 BSF single-marker parasites were grown in similar conditions to the pleomorphic EATRO1125 BSF strain but were cultured in HMI-9 medium that was not supplemented with methylcellulose. Procyclic form (PCF) parasites were cultured at 27°C in glucose-depleted SDM80 medium that contained 10% heat-inactivated FBS, 50 mM *N*-acetylglucosamine, 15 µg/mL G418, and 50 µg/mL hygromycin B. For *in vivo* infections, tsetse flies (*Glossina morsitans morsitans*) were housed at 27°C with 80% humidity on a 12 h/12 h light/dark cycle as described (7). Flies were infected according to previously standardized protocols (67, 68). Stumpy BSF parasites were fed to tsetse flies in a defibrinated horse blood meal (TCS, Buckingham, UK; catalog #: HB035) at a parasite density of 2 × 10^6^ cells/mL. Teneral flies were given a parasite blood meal 24- and 48-h post-emergence from pupae and fed thereafter at 2- to 3-day intervals so that the last blood meal was 48 h before dissection. Parasite-infected tsetse flies were dissected to harvest the insect midgut at days 3 and 7 post infection. Parasites were isolated from the tsetse midgut from independent replicates and lysed in solution D (69). RNA was isolated and converted to cDNA for qRT-PCR analysis in this study and used for RNA-Seq analysis in another study (7).

### DNA constructs used for generating transgenic cell lines

The nucleotide sequences of all primers used to create the DNA constructs for generating the transgenic cell lines are shown in Text S1. We inserted a C-terminal PTP tag on the chromosomal allele of RESC6 (previously named MRB3010; Tb927.5.3010) using a previously created plasmid construct (70). We used a PCR-mediated *in situ* tagging method (71) to tag the chromosomal alleles of RESC13 (previously named TbRGG2; Tb927.10.10830) using pPOTv4 plasmid as a template. The pPOTv4 template plasmids provide drug-resistance cassettes for puromycin (MHT tagging). To generate a C-terminal *in situ* tagging PCR amplicon for RESC13, the forward and reverse primers harbored 5′ flanking regions corresponding to the last 80 nucleotides (nt) of the *RESC13* ORF (excluding the stop codon) and the reverse complement of the first 80 nt of the *RESC13* 3′UTR, respectively. To generate the RDK1, RDK2, and p22 RNAi cell lines, RNAi amplicons were PCR-generated using *T. brucei* EATRO1125 genomic DNA as the template. The primers were designed such that the resulting PCR amplicons harbored the restriction sites for BamHI and HindIII on their 5′ and 3′ ends, respectively. The PCR amplicons were subsequently subcloned into the p2T7-177 RNAi vector between the BamHI and HindIII restriction sites (72). The p2T7-177_RDK1_, p2T7-177_RDK2_, and p2T7-177_p22_ plasmids were NotI-digested before transfection as previously described (72).

### Parasite transfection and selection

For all cell lines generated in this study, we transfected the pleomorphic slender BSF and acquired positive transfectants. To obtain the corresponding PCF cell line, the slender BSF cell line was differentiated *in vitro* to the PCF as described in the next subsection. Approximately 4 × 10^7^ slender BSF parasites were suspended in 100 µL of electroporation buffer (73) containing 10 µg of the appropriate DNA construct and electroporated with the Lonza Nucleofector 2b device using the X-001 protocol. After electroporation, parasites were immediately transferred to 100 mL of pre-warmed modified HMI-9 medium supplemented with 15% FBS and 0.25% methylcellulose. Transfected parasites were allowed to rest for 12–16 h at 37°C with 5% CO_2_. The transfectant culture was diluted 1:2, 1:5, 1:10, and 1:100 with fresh modified HMI-9 medium containing the appropriate selection drugs and aliquoted into 24-well plates (74). For the selection of the RDK1, RDK2, and p22 RNAi cell lines, phleomycin was added to a final concentration of 1.5 µg/mL. For the selection of the RESC6-PTP and RESC13-MHT cell lines, puromycin was added to a final concentration of 0.5 µg/mL. Individual wells were monitored daily for the outgrowth of drug-resistant clones to prevent transfectants from proliferating beyond 1 × 10^6^ cells/mL. Outgrowing clones were then subsequently cultured in a modified HMI-9 medium containing 10% FBS, 1.1% methylcellulose, and the appropriate selection drug. Slender BSF transfectants were verified before differentiation to PCF. Once slender BSF cell lines were differentiated to PCF, the RNAi cell lines were maintained in 2.5 µg/mL phleomycin, and the RESC6-PTP and RESC13-MHT cell lines were maintained in 1 µg/mL puromycin.

### *In vitro* differentiation of BSF to PCF

To differentiate slender BSF to PCF, we resuspended slender BSF in fresh modified HMI-9 medium at a cell density of 5 × 10^5^ cells/mL. Slender BSF parasites were allowed to grow without dilution for 48 h to promote cell density-dependent differentiation to the stumpy BSF. Stumpy BSF parasites were resuspended in chilled (~21°C) SDM80 medium supplemented with 50 mM *N*-acetylglucosamine, 20 mM glycerol, 3 mM citrate, and 3 mM *cis*-aconitate at a cell density of 2 × 10^6^ cells/mL and cultured at 27°C with 5% CO_2_ for 24 h (75, 76). After 24 h, the differentiating parasites were transferred to fresh SDM80 medium without the addition of citrate and *cis*-aconitate at a cell density of 1 × 10^6^ cells/mL for an additional 48 h (72 h total) to complete differentiation and outgrowth as procyclics.

### SDS-PAGE and Western blotting

To prepare whole cell lysates, 5 × 10^7^ cells were harvested by centrifugation, washed once in 1× phosphate-buffered saline (PBS), and lysed in 150 µL of whole cell lysis buffer [20 mM Bis-Tris pH 7.5, 100 mM NaCl, 10 mM EDTA pH 8.0, 1.5% (vol/vol) Triton X-100, cOmplete Protease Inhibitor Cocktail (Roche), and 0.1% (wt/vol) glycerol]. Cell lysates were allowed to incubate on ice for 5 min before the addition of 75 µL of NuPAGE LDS Sample Buffer (Thermo Fisher; Cat. No: NP0007) and 25 µL of 10× NuPAGE Sample Reducing Agent (Thermo Fisher; Cat. No: NP0004). The completed samples were heated at 95°C for 5 min. For SDS-PAGE, 10 µL of each whole cell lysate sample (~2 × 10^6^ cell equivalents) was loaded on a NuPAGE 4%–12% Bis-Tris Mini Protein gel (Thermo Fisher; Cat. No: NP0335BOX). Proteins were transferred to a nitrocellulose membrane, blocked with 5% nonfat milk in 1× Tris-buffered saline (TBS), and probed with primary antibodies diluted in 1× TBS containing 0.25% Tween-20 (TBST). We used Goat Anti-Mouse StarBright Blue 520 and Goat Anti-Rabbit StarBright Blue 700 (Bio-Rad; Cat. No: 12005866 and 12004161) as secondary antibodies (Bio-Rad) for protein detection on a ChemiDoc Imaging System (Bio-Rad). The list of primary antibodies and their corresponding sources and dilutions are listed in Text S1.

### RNA isolation, luciferase spike-in control, and quantitative RT-PCR analysis

We isolated total RNA from ~2 × 10^8^ parasites by lysing in 1 mL of TRIzol (Ambion). To serve as a spike-in control, 2 µL of luciferase spike-in dilution (1 ng/mL luciferase RNA) was added to the 1 mL of TRIzol-cell lysate for a final concentration of 1 ng per 1 × 10^8^ cells. To prepare the luciferase spike-in dilution, 900 µL of TRIzol, 99 µL of whole cell lysis buffer, and 1 µL of luciferase control RNA (1 mg/mL) (Promega; Cat. No: L4561) were mixed, divided into 40 µL aliquots (to avoid excessive freeze-thawing), and stored at −80°C. After spiking with luciferase RNA, total RNA was extracted from the TRIzol lysate using chloroform, precipitated using ammonium acetate and ethanol, and resuspended in RNase/DNase-free water. Approximately 30 µg of total RNA was rigorously treated with 6 U of rDNaseI (Ambion) at 37°C for 2 h to remove DNA contamination. For qRT-PCR analysis, 1 µg of DNase-treated RNA was converted to cDNA using iScript reverse transcriptase and random hexamer primers (Bio-Rad). Real-time PCR amplification and detection were performed on a CFX Real-Time System thermocycler (Bio-Rad), and data were analyzed using the CFX Maestro software (Bio-Rad). Primers specific for total and/or edited COI, COII, COIII, CYb, ND4, ND8, A6, RPS12, MURF2, and edited CR4 were previously published (29, 41, 45, 50). Primers specific for 5′-edited COIII, total ND7, edited ND7-5′, edited ND7-3′, total CR4, RDK1, and RDK2 were designed for this study and are listed in Text S1. For all qRT-PCR analyses, we used three biologically independent replicates (with three technical replicates for each biological replicate). Values were normalized relative to the luciferase RNA spike-in signal.

### Droplet digital PCR

The DNase-treated RNA isolated from the three biological replicate sets of slender BSF, stumpy BSF, differentiating, and PCF parasites used for qRT-PCR analysis in Fig. 1 was also used for ddPCR. For each transcript analyzed with ddPCR, gene-specific cDNA was generated from 0.5 µg of DNase-treated RNA using iScript Select cDNA Synthesis Kit (Bio-Rad). PCR mixtures of 25 µL were created consisting of the following: 12.5 µL of 2× EvaGreen ddPCR supermix (Bio-Rad), 2.5 µL of forward primer (1 µM), 2.5 µL of reverse primer (1 µM), 6.5 µL of water, and 1 µL diluted cDNA. Twenty microliters of the PCR mixture and 70 µL of droplet generation oil were loaded onto a Bio-Rad DG8 cartridge. Droplets were created using a QX200 droplet generator from Bio-Rad. Droplets were loaded into a Bio-Rad ddPCR 96-well plate, and PCR was performed in a deep-well thermal cycler with the following cycling protocol: (i) 95°C for 5 min, (ii) 95°C for 30 s, (iii) annealing/extension temperature for 1 min, (iv) return to step (ii) for 39 more cycles, (v) 4°C for 5 min, and (vi) 95°C for 5 min. The optimal annealing/extension temperatures for each transcript were determined prior to quantification and listed in Text S1. After PCR completion, droplets were read using a QX200 Droplet Reader (Bio-Rad). All samples were verified to have at least 10,000 accepted droplets. In most cases, the positive droplet threshold was determined automatically by QuantaSoft Analysis Pro software version 1.0.596.0525 (Bio-Rad). If the program could not determine a threshold, then the threshold was set manually. The number of copies per milliliter determined by QuantaSoft was used to calculate the copies per cell.

### RNA immunoprecipitation

The RNA immunoprecipitation (RIP) procedure is based primarily on published methods with some modifications (77). Approximately 2 × 10^9^ parasites were harvested by centrifugation and washed once in 10 mL of either pre-warmed (37°C), FBS-free, and methylcellulose-free HMI-9 medium (for slender and stumpy BSF parasites) or 10 mL of pre-warmed (27°C) and FBS-free SDM80 medium (for CCA-treated, differentiating and PCF parasites). The cells were resuspended in 50 mL of ice-cold, FBS-free HMI-9 or SDM80 medium and split between two 145 × 20 mm Petri dishes (~25 mL each). Cells were UV-irradiated once at 400 mJ/cm^2^ at 254 nm. Cells were centrifuged and washed once in cold 1× PBS. We prepared crude mitochondrial fractions by resuspending the cells in 5 mL of cold SorTE buffer (600 mM sorbitol, 20 mM Tris-HCl pH 8.0, 2 mM EDTA) and subsequently adding 5 mL of cold SorTE buffer containing 0.05% (wt/vol) digitonin. The 10 mL cell suspension was gently mixed by inverting the tube three times and then was incubated on ice for 5 min. The cell suspensions were centrifuged at 6,800 × *g* for 5 min at 4°C, and the resulting crude mitochondrial pellets were flash frozen in liquid nitrogen and stored at −80°C until ready to use. Mitochondrial pellets were thawed and resuspended in 4 mL of cold lysis buffer [10 mM Bis-Tris pH 7.5, 100 mM NaCl, 10 mM EDTA, 1% (vol/vol) Triton X-100, 200 U/mL Superase-In RNase inhibitor (Thermo Fisher), and cOmplete protease inhibitors]. Complete lysis was achieved by incubating for 30 min at 4°C with constant rocking. The lysates were clarified by centrifugation at 20,000 × *g* at 4°C for 20 min. Clarified lysates were mixed with 200 µL of pre-equilibrated IgG FastFlow Sepharose beads (or Superdex-200 as a negative control) and incubated for 2 h at 4°C with constant rocking.

## SUPPLEMENTAL MATERIAL

Supplemental figures and text mbio.01854-23-s0001.pdf Fig. S1 to S4 and Text S1.

ASM does not own the copyrights to Supplemental Material that may be linked to, or accessed through, an article. The authors have granted ASM a non-exclusive, world-wide license to publish the Supplemental Material files. Please contact the corresponding author directly for reuse.
