# Supplementary material for: Developmental dynamics of mitochondrial mRNA abundance and editing reveal roles for temperature and the differentiation-repressive kinase RDK1 in cytochrome oxidase subunit II mRNA editing
Source: mBio. 2023 Oct 5;14(5):e01854-23. doi: 10.1128/mbio.01854-23 (PMC10653865; doi:10.1128/mbio.01854-23)
Supplement: Supplemental figures and text — Fig. S1 to S4 and Text S1. [file mbio.01854-23-s0001.pdf]

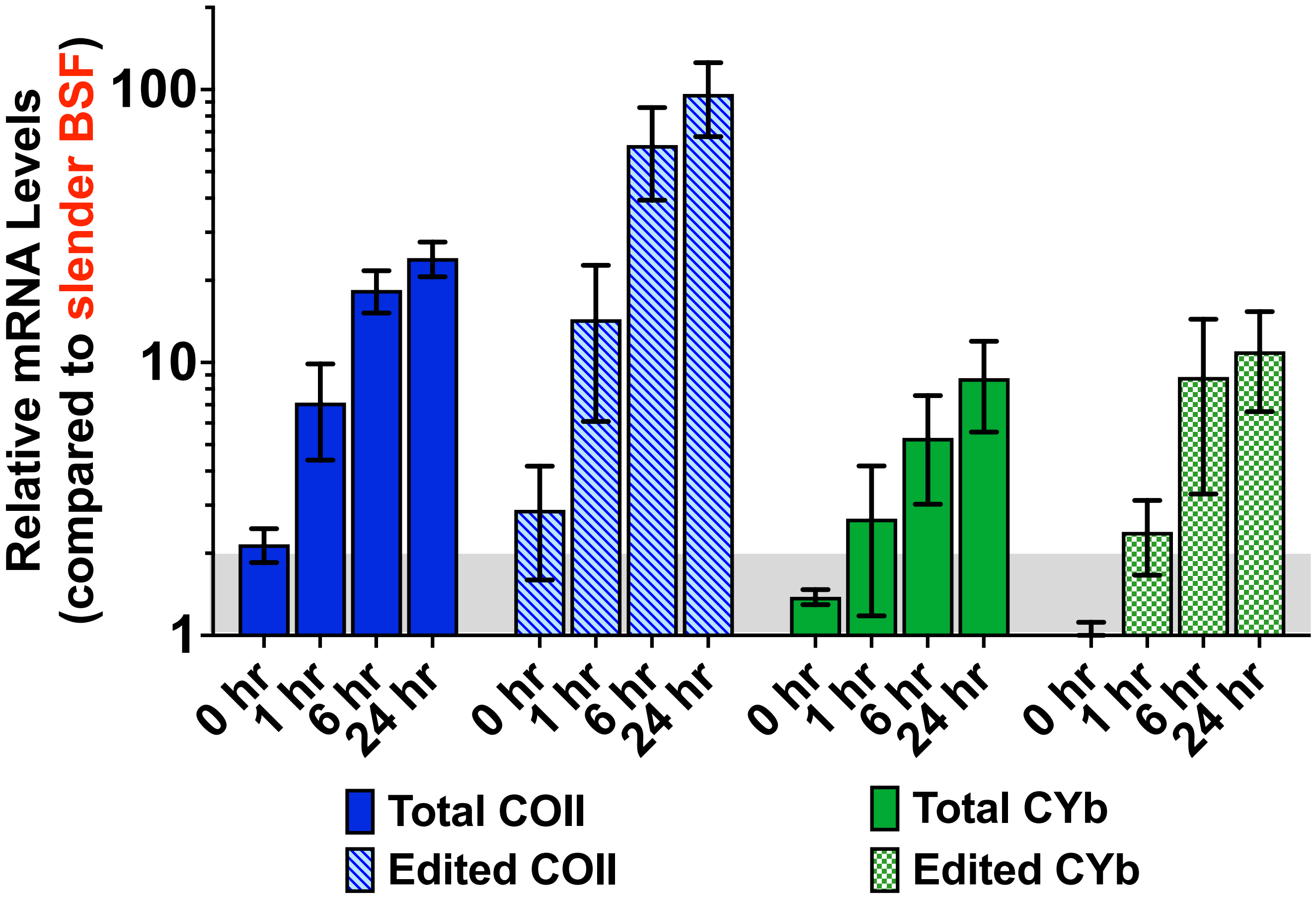

Fig. S1. Time kinetics of the abundance of total and edited COII and CYb mRNAs during BSF-to-PCF differentiation. Quantitative RT-PCR analysis of total/edited COII and CYb mRNAs as stumpy BSF are stimulated to differentiate to PCF *in vitro* after 1, 6, and 24 hours. Resting, unstimulated stumpy BSF (0 hr) are compared to slender BSF are shown for comparison.

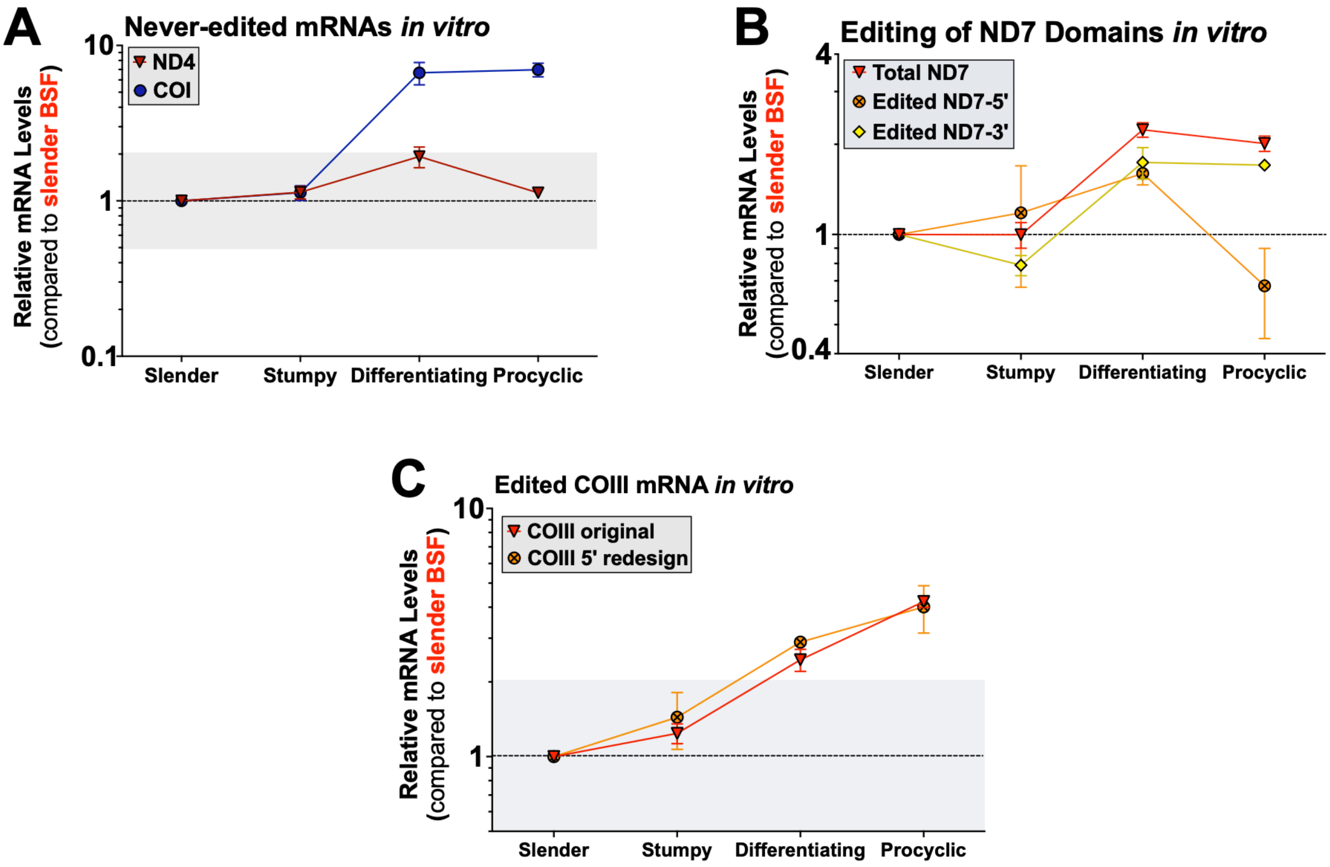

Fig. S2. **(A)** Quantitative RT-PCR analysis of the never-edited mitochondrial mRNAs ND4 and COI as slender BSF differentiate to PCF *in vitro*. **(B)** Quantitative RT-PCR analysis distinguishing the distinct editing of the 5' and 3' domains of ND7 mRNA as slender BSF differentiate to PCF. **(C)** Quantitative RT-PCR analysis of edited COIII mRNA from slender BSF to PCF using two sets of primers. The original edited COIII primers (50) are compared with results from redesigned edited COIII primers that detect editing at the extreme 5' of edited COIII mRNA.

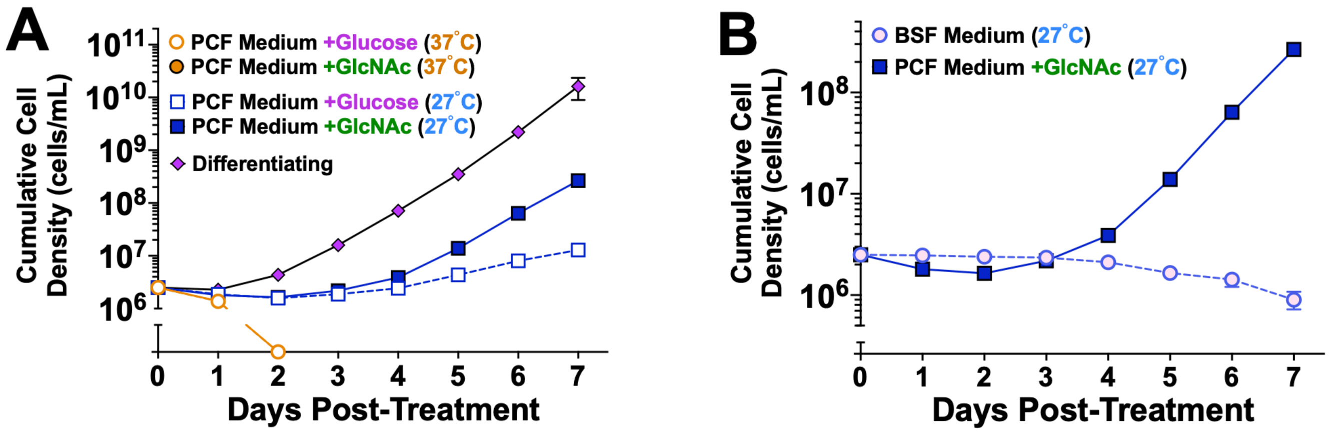

<u>Fig. S3</u>. Growth kinetics of outgrowing parasites after stumpy BSF are recultured under different conditions. (A) Effect of temperature and glucose concentration on the outgrowth of PCF parasites after stumpy BSF are shifted to PCF medium with 5.55 mM glucose (+Glucose) or 50 mM N-acetyl-D-glucosamine (+GlcNAc) at 37°C or 27°C in the absence of 3 mM citrate/3 mM cis-aconitate (CCA). The outgrowth kinetics of PCF parasites from stumpy BSF parasites that received the complete *in vitro* differentiation signal that consists of PCF medium supplemented with 50 mM GlcNAc and CCA at 27°C (Differentiating) are shown for comparison. **(B)** Outgrowth kinetics of PCF parasites from stumpy BSF cultured in BSF medium at 27°C. The outgrowth kinetics of PCF parasites from stumpy BSF cultured in PCF medium with 50 mM GlcNAc at 27°C is shown for comparison.

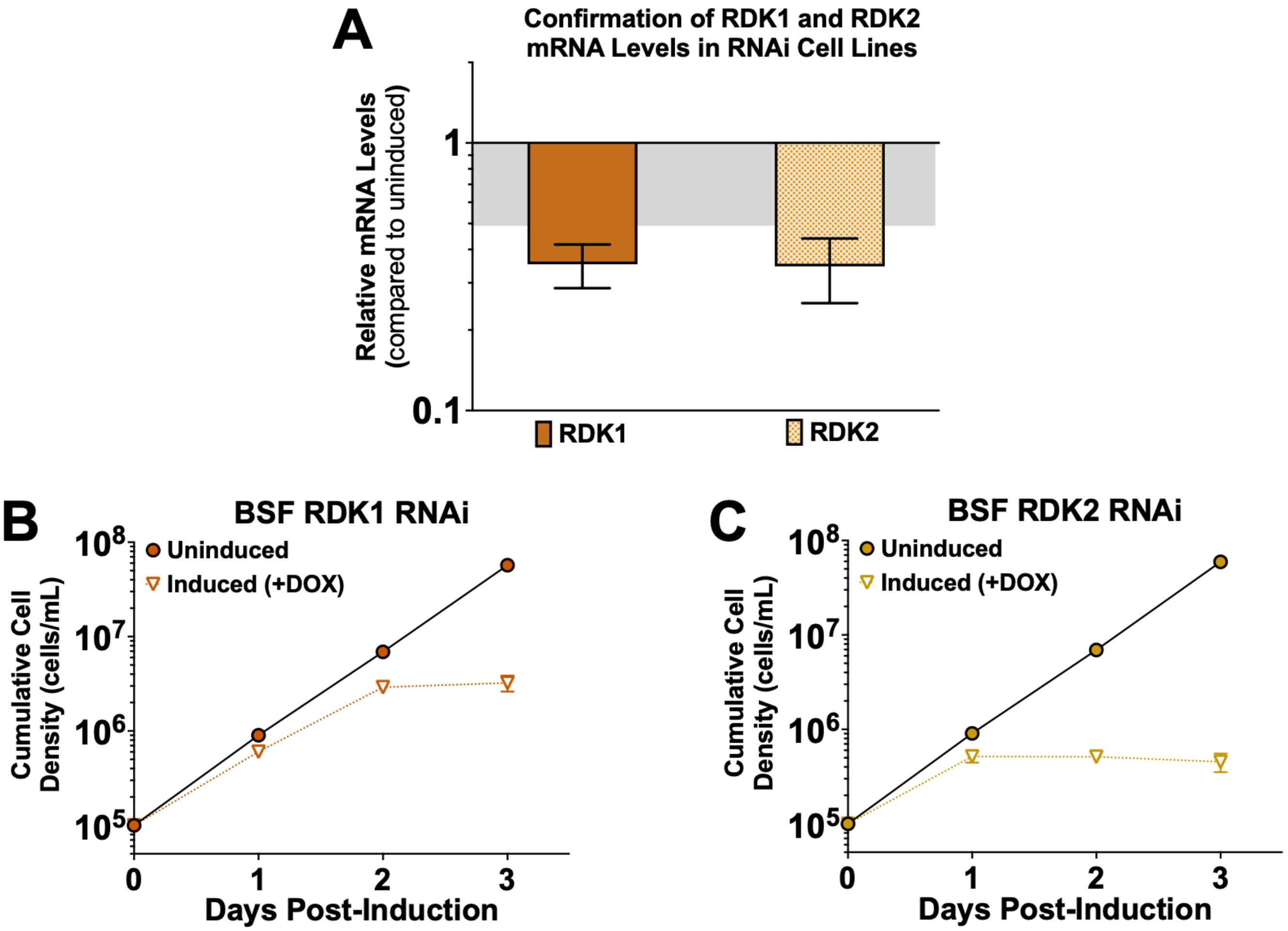

Fig. S4. Confirmation of BSF RDK1 RNAi and RDK2 RNAi cell lines. (A) Quantitative RT-PCR analysis to confirm RDK1 and RDK2 mRNA levels in the respective RNAi cell lines after induction with doxycycline (0.5 μg/mL) for 24 hours. (B-C) Growth kinetics of BSF RDK1 RNAi and BSF RDK2 RNAi cell lines in the absence (uninduced) or presence of doxycycline (induced +DOX) for up to 3 days.

TEXT S1.

A. Electroporation buffer for trypanosome transfection

The 3X stock buffer consists of 200 mM disodium phosphate (Na_2HPO_4), 70 mM monosodium phosphate (NaH_2PO_4), 15 mM potassium chloride, and 150 mM HEPES pH 7.3. The stock is filter-sterilized with a 0.22-micron filter and stored at 4°C. To make a working stock of 1X electroporation buffer, mix 600 μL of sterile water, 350 μL of 3X stock buffer, and 100 μL of 1.5 mM calcium chloride.

B. Antibody dilutions and sources

Antibody target	Species	Dilution	Source
VSG (AnTat1.1)	Rabbit	1:10000	Dr. James Bangs, University at Buffalo
EP1 procyclin	Rabbit	1:10000	Dr. James Bangs, University at Buffalo
GPEET	Rabbit	1:1000	Dr. Isabel Roditi, University of Bern
PAD1	Rabbit	1:2000	Dr. Keith Matthews, University of Edinburgh
NDUFA	Rabbit	1:1000	Dr. Alena Zíková, Czech Academy of Sciences
Rieske/RISP	Rabbit	1:1000	Dr. Alena Zíková, Czech Academy of Sciences
COIV	Rabbit	1:1000	Dr. André Schneider, University of Bern
ATPase sub β	Rabbit	1:2000	Dr. Alena Zíková, Czech Academy of Sciences
TAO	Mouse	1:250	Dr. Minu Chaudhuri, Meharry Medical College
EF1α, CBP-KK1	Mouse	1:5000	Sigma-Aldrich, Catalog #: 05-235

C. Primer nucleotide sequences

**Amplicon target**	**Purpose**	**Sequence (5' to 3')**
5' Edited COIII forward	qRT-PCR	CTACCAAACAATAAACAAATAAATCCCAAC
5' Edited COIII reverse	qRT-PCR	ATGTTTTTGTTTCGTTGTATATTTGTTGGTGTTAG
Total ND7 forward	qRT-PCR	CATGACTACATGATAAG
Total ND7 reverse	qRT-PCR	CTTTTCTGTACCACGATGC
Edited ND7-5' forward	qRT-PCR	GTATCATTTTATGTTATTTTTGGTAG
Edited ND7-5' reverse	qRT-PCR	CCTATAATACAATCAATATAAAC
Edited ND7-3' forward	qRT-PCR	GGTTAGATTATGTTAGTGTTTGTTG
Edited ND7-3' reverse	qRT-PCR	CAAACCATTAAAACTACGGGTAAATTC
Total ND8 forward	qRT-PCR	GAATCAATTTAATAATTTTAAGTTTTGG
Total ND8 reverse	qRT-PCR	CCCGCCTCTCTGGTTCTCTGGGAAATC
Total CR4 forward	qRT-PCR	TAATTTATTGTTATCTTTGTGTATTTATTAAAAAGGGGC
Total CR4 reverse	qRT-PCR	CTTTCCTCATAGATCAATCCCAACCAAAC
RDK1 forward	qRT-PCR	GGAGATGGAAAAGCATCG
RDK1 reverse	qRT-PCR	CGACTCCTTCAAGAAAATC
RDK2 forward	qRT-PCR	GTGGGCGTAATATGGATGAGC
RDK2 reverse	qRT-PCR	GCCACGGCGAATGAAAGGTAG
RDK1 forward	RNAi plasmid	GATCGATCGGATCCGGAAATAATTCTGTGGTGTTAGGTGCGTAC
RDK1 reverse	RNAi plasmid	GATCGATCAAGCTTCCAACTGTTCAAAATAACAATATCCCTCGA
RDK2 forward	RNAi plasmid	GATCGATCGGATCCGGGTCAGGGGAGTTTTGGTAGCGCATGGCG
RDK2 reverse	RNAi plasmid	GATCGATCAAGCTTCGGGGCTCAAATAATATGGCGTCCCACAGA
p22 forward	RNAi plasmid	GATCGGATCCCGTAAAGAGGAGAGGAGGGGGGGGGC
p22 reverse	RNAi plasmid	GATCAAGCTTCGCATCATCGCCCCTCCCTTGTCCTTC

RESC13-MHT forward (*in situ* tagging)
GGGAGCAGGCGATAGCGGAAGGAGATTGTAGCGCGCCCGCGCCTCCCGCCGAAGTGGCAGGTGATGCCAGTCAGA
AGGTGGGTTCTGGTAGTGGTTCC

RESC13-MHT reverse (*in situ* tagging)
TTATTTACCCGCTTGACGCCAGTTTCCAGCGCTTGGCGGAACGGGGTGGGTATGCTACTAATATTCCCGTAAATAGG
GTTCCAATTTGAGAGACCTGTGC
